# Versatile DNA‐Functionalized Biohybrid Hydrogel Platforms for Electrothermally Activated On‐Demand Payload Release

**DOI:** 10.1002/advs.75573

**Published:** 2026-05-07

**Authors:** Mengqiu Sun, Zhenshuai Tang, Jia Zhang, Rui Song, Wei Luo, Juan Huang, Jincheng Zeng, Zhen He, Hao Zhang

**Affiliations:** ^1^ Guangdong Provincial Key Laboratory of Mathematical and Neural Dynamical Systems Great Bay University Dongguan Guangdong China; ^2^ Department of Materials Science and Engineering Southern University of Science and Technology Shenzhen Guangdong China; ^3^ Dongguan Key Laboratory of Medical Bioactive Molecular Developmental and Translational Research Guangdong Provincial Key Laboratory of Medical Immunology and Molecular Diagnostics Guangdong Medical University Dongguan China

**Keywords:** biohybrid materials, cancer therapy, chronic wound healing, DNA nanotechnology, neural modulation, on‐demand drug delivery

## Abstract

Current drug delivery systems struggle to achieve precise control, rapid response, and high biocompatibility. Addressing these challenges, we developed an electrothermally responsive biohybrid hydrogel incorporating DNA‐functionalized nanostructures for on‐demand payload release. Unlike conventional platforms relying on passive diffusion or biochemical triggers, our system utilizes electrical stimulation to produce localized heating that simultaneously alters DNA conformation and hydrogel structure. This dual mechanism enables rapid electrically controlled release within seconds while preserving excellent biocompatibility. The modular DNA design permits easy customization through sequence modifications, enabling flexible adaptation for diverse therapeutic applications, including precision oncology, neural regulation, and chronic wound healing.

## Graphical abstract

A DNA‐functionalized biohybrid hydrogel is developed by integrating Cu‐mediated nucleic acid microflowers into a thermoresponsive matrix, enabling electrothermally triggered, on‐demand payload release. The platform offers spatiotemporally controllable therapeutic delivery with promising applications in precision oncology, neural regulation, and chronic wound healing.

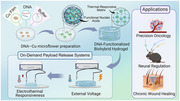

## 1 Introduction

Molecular release systems with precise control are critical for numerous applications, including biomedical therapies, smart materials, and precision catalysis. These applications require advanced materials capable of delivering unmatched precision, instant activation, and optimal biocompatibility [1, 2, 3, 4, 5]. While current stimulus‐responsive platforms offer fundamental on‐demand release, they exhibit certain inherent limitations. Enzyme‐ or pH‐triggered systems are restricted by their dependence on specific biochemical environments, compromising external real‐time control [6, 7, 8]. Photoresponsive materials, despite their remarkable targeting accuracy, encounter difficulties with insufficient tissue penetration and potential light‐induced toxicity [9, 10]. Electrical stimulation represents a promising alternative because of its noninvasive nature, tunability parameters, and ultrafast response characteristics [11, 12]. Existing electro‐responsive platforms mostly rely on redox reactions or ionic transport mechanisms, which typically exhibit delayed release kinetics and reduced biocompatibility [13, 14, 15, 16, 17]. Significantly, the Joule heating effect opens new possibilities for next‐generation systems, though demanding enhanced capabilities from thermally responsive components [18, 19]. Conventional thermos‐responsive hydrogels display temperature‐dependent phase transitions, but their requirement for external heating limits site‐specific control, and simple mechanical compression fails to achieve molecular‐level release precision [20, 21, 22, 23]. Therefore, the key challenge in advancing controllable release systems centers on effectively integrating the advantages of electrical control with thermal response mechanisms, while incorporating intelligent molecular release triggers [24, 25, 26, 27].

**FIGURE 1:**
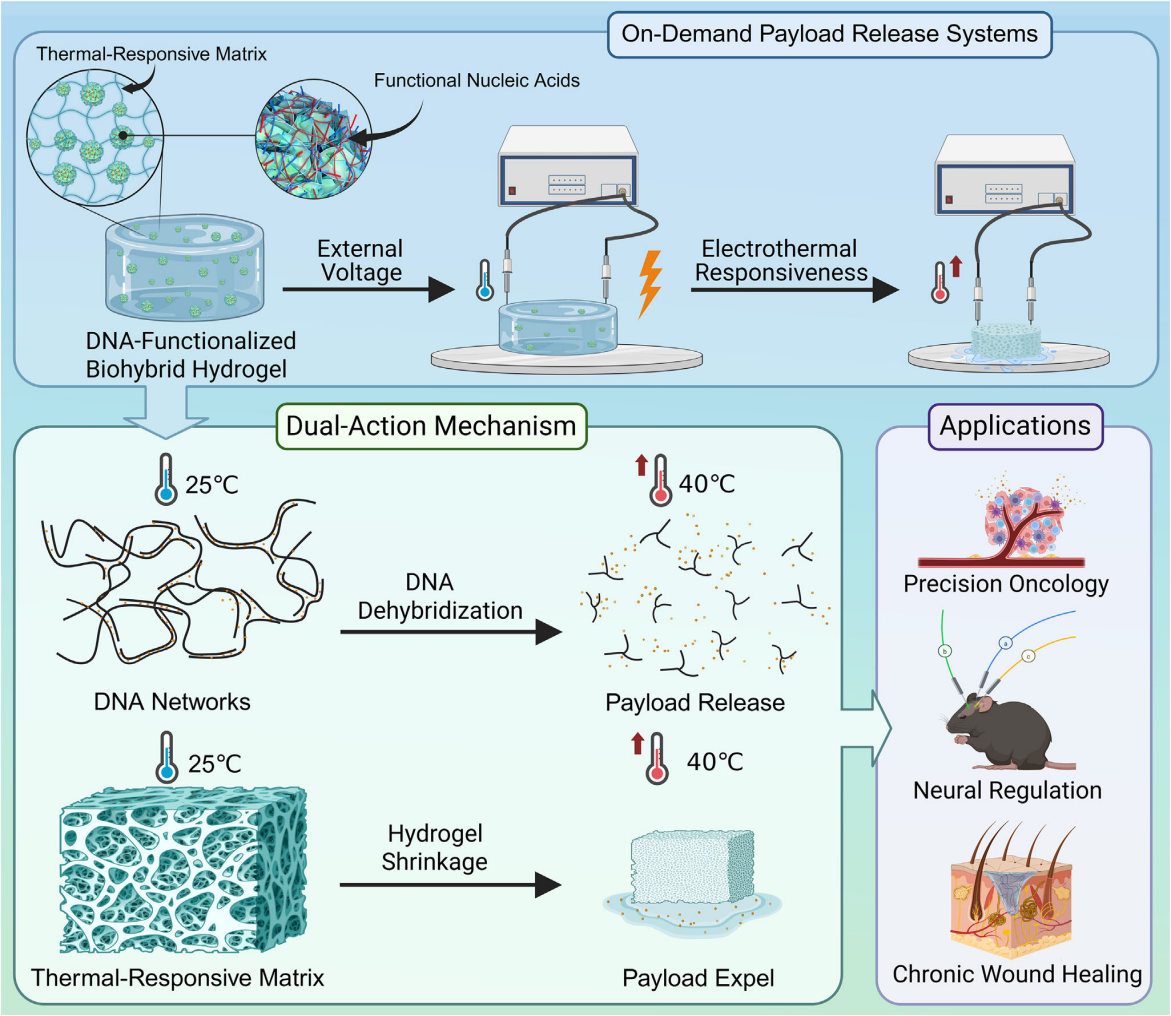
Schematic illustration of the electrothermally activated on‐demand payload release mechanism of DNA‐functionalized biohybrid hydrogel.

Here, we present a novel controlled‐release platform combining electrical stimulation, thermal response, and molecular structural changes for achieving on‐demand payload release via a well‐coordinated multiscale process (Figure 1). The system uses functional units of DNA‐modified copper microstructures, in which optimized DNA sequences and copper ion coordination form microflowers with layered, petal‐like architectures. These 3D structures provide high payload capacity and can efficiently convert electrical energy into heat. The system's principal innovation resides in its unique mechanism of electrical stimulation, where controlled heating simultaneously triggers both molecular‐scale conformational changes in DNA motifs and macroscopic hydrogel contraction. When a mild electric field is applied, the DNA‐copper microflowers produce localized heating through the Joule effect. This heating initiates two parallel release processes. At the molecular level, temperature‐sensitive DNA structures unfold reversibly, releasing encapsulated functional molecules into the surrounding hydrogel matrix. At the same time, the thermosensitive hydrogel shrinks, creating mechanical pressure to expel both water and payload. This dual‐action mechanism ensures precise payload release while reducing unintended leakage.

To show the system's wide range of uses, we implemented it in three representative biomedical applications. First, for cancer therapy, we developed an electrically responsive drug delivery platform by loading doxorubicin onto AT‐rich double‐stranded DNA, achieving targeted tumor cell treatment. Second, in neural modulation, we engineered a programmable neurotransmitter release system using dopamine‐specific DNA aptamers, enabling precise neural regulation at the cellular level. Third, we applied the system to diabetic wound healing, where electrically triggered antibiotic release significantly accelerated tissue regeneration in vivo. These implementations collectively showcase the platform's capacity as a multifunctional tool for controlled molecular delivery, with broad potential in precision medicine, bioelectronic therapies, and regenerative medicine.

## 2 Results and Discussion

### 2.1 Synthesis and Characterization of DNA‐Functionalized Microflowers for Hydrogel Integration

**FIGURE 2:**
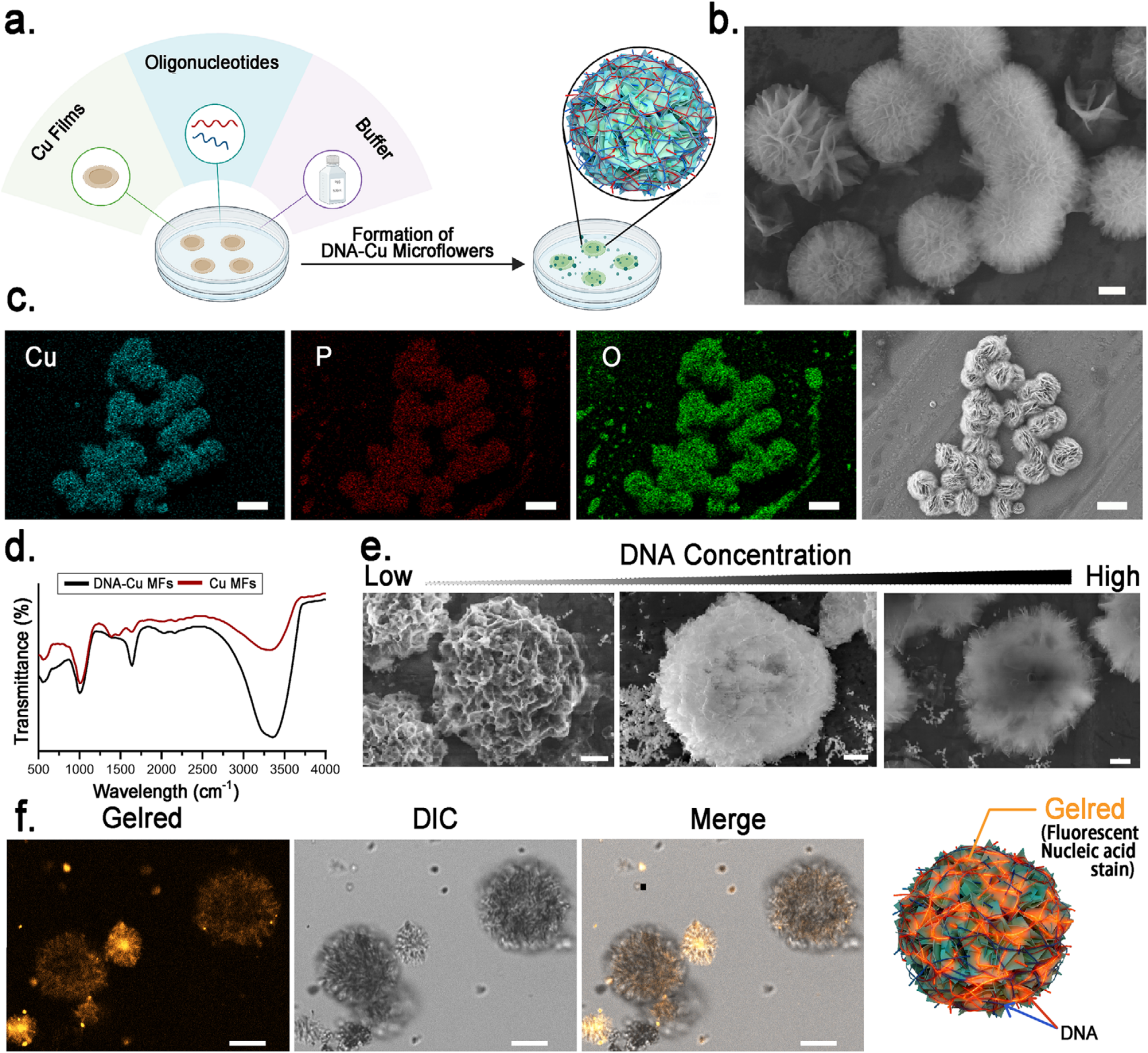
Fabrication and structural analysis of DNA‐copper microflowers for hydrogel integration. (a) Schematic representation of microflowers assembly processes mediated by DNA‐copper coordination. (b) High‐resolution SEM image of the hierarchical petal‐like architecture (scale bar: 1 µm). (c) Elemental composition analysis showing co‐distribution of Cu (cyan), P (red), and O (green) (scale bar: 20 µm). (d) FTIR spectral comparison identifying DNA‐specific vibrational signatures (black: DNA‐copper microflowers; red: copper microflowers). (e) SEM images showing morphological evolution with increasing DNA concentrations from 0 to 0.5 µM (scale bars: 5 µm). (f) Confocal microscopy validation of DNA integration using a fluorescent nucleic acid‐specific dye (staining with GelRed, scale bar: 10 µm).

The fabrication and structural characterization of DNA‐copper microflowers (DNA‐Cu MFs) form the essential basis of this study. We established a preparation approach that coordinates copper ions with pre‐assembled DNA structures using copper sheets as the starting material. We chose copper ions as the building block because their specific coordination chemistry with phosphate groups allows for the formation of high‐surface‐area structures under mild conditions that are difficult to achieve with other metal ions like calcium or magnesium [28, 29, 30]. Furthermore, in hydrated environments, this copper‐based hybrid architecture shows a superior capacity for electrothermal conversion [31], enabling the rapid temperature elevation necessary for our dual‐response mechanism, which clearly distinguishes it from many alternative metal‐integrated biomaterials. Through rational sequence design, customized DNA building blocks were first annealed to form structural units, which were then incorporated during copper phosphate crystallization, enabling stable DNA attachment to microflower surfaces through coordination bonds and electrostatic effects (experimental detail provided in Supporting Information). The obtained hybrid microstructures with well‐defined morphologies (Figure 2a) were isolated through centrifugation to ensure phase purity before subsequent characterization.

Systematic investigation of reaction conditions revealed that DNA concentration significantly affects the morphology of the microflowers. As shown in Figure 2e, increasing DNA concentration led to microflowers with thinner and more elongated petal‐like features, showing that DNA not only guides the nucleation of copper ions through its phosphate backbone but also facilitates the formation of organized, multilayered structures. Optimized microflower morphology with spherical shape and uniform size distribution was achieved at 0.01 m PBS concentration, whereas elevated buffer levels disrupted the crystalline organization (Figure S1). Within the sodium chloride range of 0.1–0.5 m, chloride ions effectively modulated copper dissolution and recrystallization processes. Optimal microflower formation occurred at 0.3 m NaCl, yielding structurally uniform particles with minimal byproducts (Figure S2).

Scanning electron microscopy (SEM) analysis showed that the microflowers prepared under optimal conditions display a characteristic petal‐like morphology with diameters of 5–10 µm, consisting of interconnected sheet‐like structures about 20 nm thick (Figure 2b). Elemental mapping analysis (Figure 2c) confirmed the homogeneous distribution of Cu, P, and O throughout the microflower structure, while quantitative EDS measurements (Figure S3) revealed predominant copper(II) phosphate inorganic phase formation. The microflowers' composition and crystallinity were further characterized by complementary techniques, where X‐ray photoelectron spectroscopy (XPS) identified Cu^2^^+^‐phosphate coordination (Figure S4) while X‐ray diffraction (XRD) confirmed structural similarity to known copper phosphate crystals (Figure S5) [32]. Compared with pristine Cu MFs, the DNA‐Cu MFs exhibit enhanced absorption bands around 3200–3500 cm^−^^1^, attributed to O─H and N─H stretching vibrations from DNA. Notably, the characteristic phosphate vibrations at ∼1230 cm^−^^1^ (P═O) and ∼1080 cm^−^^1^ (P─O) are significantly intensified, confirming the successful incorporation of DNA. [33] The broadening and slight shifting of these peaks suggest coordination interactions between Cu^2^^+^ ions and the phosphate backbone of DNA. Additionally, confocal microscopy revealed strong red fluorescence from Gel Red‐stained DNA‐integrated microflowers (Figure 2f), with control experiments confirming the dye's specific binding to DNA bases rather than copper components (Figure S6), demonstrating successful DNA incorporation while preserving its biological activity [34]. Concerning the conductive mechanism, experiments established that the microflowers show negligible conductivity when dry (Figure S7). The hydrogel system's conductivity likely originates from both hydrated copper phosphate microflowers and mobile ions within the gel matrix, collectively enabling electrothermal conversion.

This distinctive preparation method enables regulated assembly of organic–inorganic hybrid microstructures via interaction between DNA strands and copper ions. DNA acts not just as a template to control morphology but also engages directly in ion coordination through its nucleobases and phosphate groups. The resulting 3D organized structure maintains copper phosphate's physicochemical properties while providing the system with DNA's unique molecular recognition and loading abilities, creating the basis for developing biocompatible electrically responsive delivery systems.

### 2.2 Construction and Electrothermal Properties of Biohybrid Hydrogels

**FIGURE 3:**
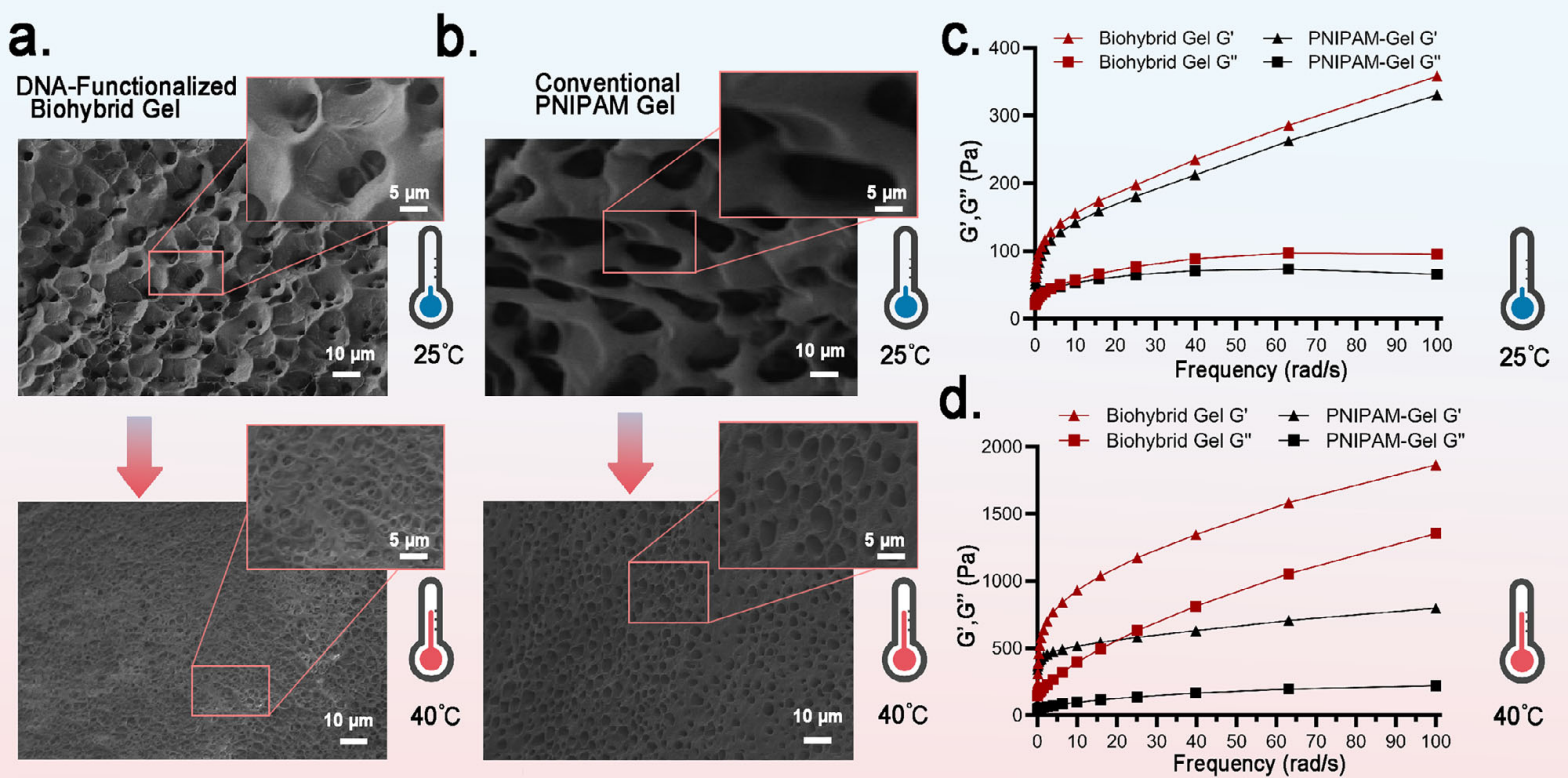
Morphological and mechanical properties of the DNA‐functionalized biohybrid hydrogel. (a,b) SEM images showing the temperature‐dependent morphological transition of DNA‐functionalized biohybrid hydrogel (a) and conventional PNIPAM hydrogel (b) at 25°C (top) and 40°C (bottom), respectively. (c,d) Rheological measurements demonstrate the storage (*G′*, triangle) and loss (*G′′*, square) moduli of DNA‐functionalized biohybrid hydrogel (red) and conventional PNIPAM hydrogel (black) under frequency sweep at 25°C (c) and 40°C (d).

The performance of the hybrid hydrogel critically depends on its electrothermal response properties, which directly determine three key system parameters: response speed, payload release accuracy, and operational reliability. Our optimized fabrication methods involve ultrasonic dispersion of pre‐synthesized DNA‐copper microflowers within a N‐isopropylacrylamide precursor solution. Subsequent low‐temperature polymerization was initiated through an ammonium persulfate/tetramethylethylenediamine (APS/TEMED) redox system with N,N‐methylenebisacrylamide crosslinking, yielding a microflower‐embedded hydrogel (MF‐Gel) that responds to both thermal and electrical stimuli. Microstructural analysis by SEM revealed uniform microflower distribution throughout the hydrogel matrix, inducing significant architectural modifications. At ambient temperature (25°C), the microflower‐embedded hydrogel displayed interconnected pores averaging 3.5 ± 1.0 µm (Figure 3a) with wall thickness approximately double that of unmodified Poly(N‐isopropylacrylamide) (PNIPAM) hydrogel (Figure 3b). Thermal treatment at 40°C caused pore contraction to 1.4 ± 0.3 µm, creating a more compact network with smoother surfaces, while regular hydrogels developed disordered macroporous structures.

Rheological analysis revealed temperature‐dependent mechanical reinforcement in the microflower‐embedded hydrogel. At 25°C, both hydrogels showed comparable storage (*G′*) and loss (*G′′*) moduli, indicating minimal reinforcement from microflowers under ambient conditions (Figure 3c). Remarkably, at 40°C, the biohybrid hydrogel demonstrated substantially enhanced mechanical properties (Figure 3d), with *G′* rapidly increasing beyond 1866 Pa compared to only ∼797 Pa for the PNIPAM hydrogel. Its *G′′* displayed frequency‐dependent growth to ∼1354 Pa, contrasting with PNIPAM's stable 221 Pa *G′′*. These properties enable effective drug release through multiple mechanisms. The elevated *G′* indicates strong microflower‐polymer interactions that maintain structural integrity during contraction, while the growing *G′′* reflects dynamic network reorganization under stimulation. Together, they ensure sustained force generation and precise release control during repeated electrical activation cycles, demonstrating the microflowers' critical role in thermally triggered payload delivery.

**FIGURE 4:**
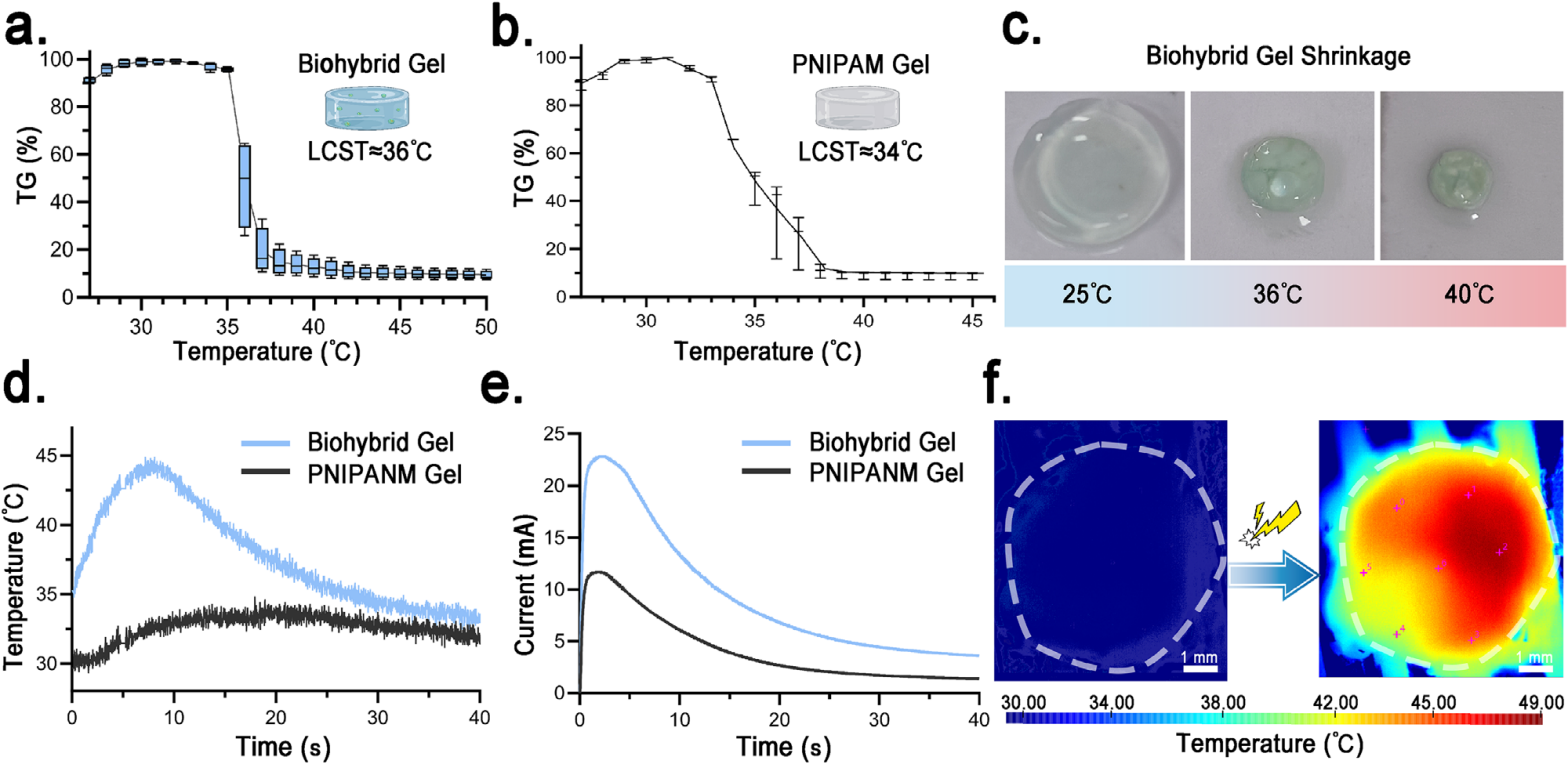
Electrothermal responsiveness of DNA‐functionalized biohybrid hydrogel. (a) Temperature‐dependent mass retention of conventional PNIPAM hydrogel. (b) Temperature‐dependent mass retention of DNA‐functionalized biohybrid hydrogel showing 90% loss occurring sharply at 35°C–37°C. (c) Visualization of temperature‐induced volume transition of biohybrid hydrogel at 25°C (left), 34°C (middle) and 40°C (right). (d) Real‐time temperature monitoring of DNA‐functionalized (blue) and pure PNIPAM hydrogel (black) under 8 V applied voltage. (e) Current‐time relationship during electrical activation (blue: DNA‐functionalized biohybrid hydrogel; black: pure PNIPAM hydrogel). (f) Infrared thermal images showing localized heating distribution before (left) and after (right) electrical stimulation. Scale bars: 1 mm.

We next examined the thermoresponsive behavior of both hydrogel systems through controlled heating experiments conducted at a precisely maintained rate of 0.05°C per minute. The microflower hydrogel underwent rapid mass loss (90%) within the 35°C–37°C range, corresponding to a lower critical solution temperature (LCST) of ∼36°C (Figure 4a). This represents a ∼2°C increase compared to conventional thermosensitive hydrogels (∼34°C, Figure 4b), presumably due to microflower‐induced alterations in aqueous microenvironment organization. Moreover, visual observation clearly captured the abrupt volume contraction occurring between 25°C and 40°C, with complete phase transition achieved at ∼40°C (Figure 4c), consistent with quantitative mass loss measurements. Parallel optical density measurements revealed concomitant turbidity increases (Figure S8), which further confirms the anticipated phase transition from hydrophilic to hydrophobic states within the PNIPAM polymer networks.

The architecture of DNA‐copper microflowers enables efficient electrothermal conversion. As shown in Figure 4d (blue curve), application of 8 V electrical stimulus induced rapid temperature elevation from 30°C to ∼45°C within 7 s (heating rate ∼2.1°C/s), whereas pure PNIPAM (Figure 4d, black curve) and other control hydrogels (Figure S9) exhibited much smaller temperature changes (<0.1°C/s) under identical conditions. Current‐time curves (Figure 4e) displayed an initial ∼23 mA surge followed by progressive decay as hydrogel contraction impeded ionic conduction. Thermal imaging (Figure 4f) further showed localized heating focused near hydrogel clusters (∼49°C) compared to distal regions (∼39°C), indicating spatially selective activation capability. The charge transport mechanism appears to be predominantly mediated by ionic conduction, as evidenced by the measured conductivity value of 0.325 S/cm (Figure S10).

### 2.3 Payload Loading and Stimuli‐Responsive Release Characteristics

**FIGURE 5:**
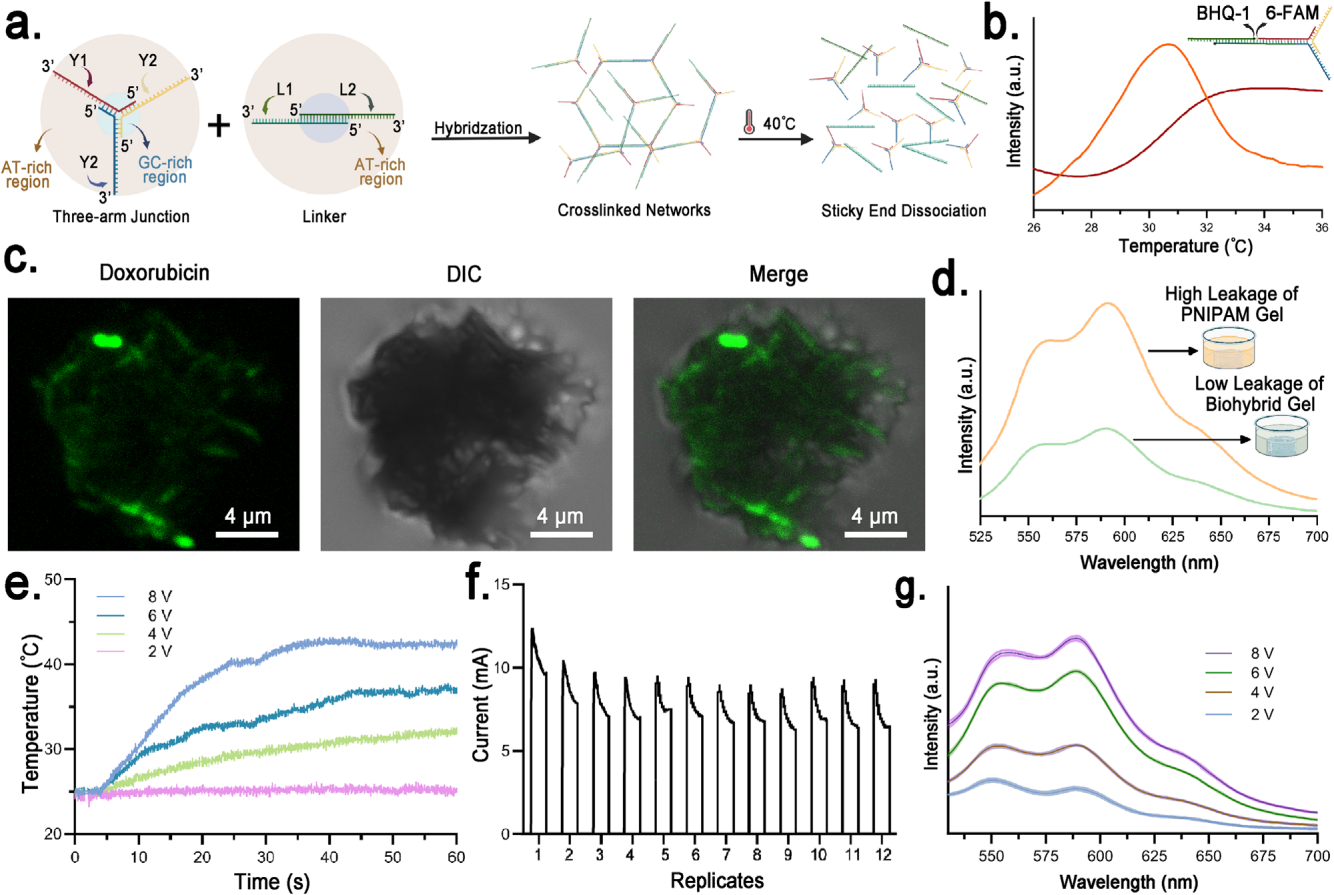
Payload Loading and controlled release characteristics of DNA‐functionalized biohybrid systems. (a) Schematic illustration of engineered DNA building blocks for temperature‐responsive dissociation and on‐demand payload release. (b) Thermal denaturation of DNA networks monitored by fluorescence (red). Derivative plot (orange) provides a precise determination of the melting transition. (c) Confocal fluorescence microscopy image showing doxorubicin distribution on microflower surfaces (Ex: 494 nm, Em: 525–700 nm, scale bar: 4 µm). (d) Fluorescence spectroscopy‐based drug release profiles of PNIPAM hydrogel (orange) and DNA‐Cu MF biohybrid hydrogel (green). The cumulative drug release was quantified using a fluorescence spectrophotometer under physiological conditions (pH 7.4, 37°C) over 2 h. (e) Temperature responses of the DNA‐functionalized biohybrid hydrogel to electrical stimulation at 2, 4, 6, 8 V. (f) Cyclic release performance of the DNA‐functionalized biohybrid hydrogel during repeated electrical stimulation (5 V, 10 s on/1 min off). (g) Fluorescence spectroscopy profiles of doxorubicin released from the DNA‐functionalized biohybrid hydrogel under electrical stimulation at different voltages.

The DNA‐integrated biohybrid system demonstrates unique dual capabilities in payload loading and controlled release. Through integration of custom‐designed oligonucleotides as active elements, we developed a tunable platform that can be adapted for different medical uses through variation of nucleotide sequences. As proof of concept, we designed a hybrid DNA network composed of a three‐arm junction and a complementary double‐stranded segment (Figure 5a). The Y‐shaped three‐arm building block features two distinct sequence domains: a GC‐rich core region (GC content = 92.3%) that forms a thermodynamically stable three‐arm junction, and an AT‐rich sticky end region (AT content = 100%) that enables thermally programmable drug liberation due to its sharp melting properties. The complementary double‐stranded segment consists of a 15‐base‐pair duplex domain and an AT‐rich overhang that is fully complementary to the sticky‐end of the Y‐shaped structure (Figure S11). Upon annealing, the sticky ends of the two components precisely hybridize, forming an extended duplex assembly with both structural stability and thermal responsiveness. The DNA architecture is likely associated with copper microflowers through phosphate coordination, maintaining structural stability while preserving biocompatibility and drug‐loading efficiency. The AT‐rich termini serve as thermal switches, reversibly unfolding above their melting point to enable on‐demand payload release.

Temperature‐dependent fluorescence analysis was performed to characterize the thermal‐responsive behavior of the engineered DNA assembly. The Y1 strand of the Y‐shaped structure was labeled with a FAM fluorophore, while the complementary duplex terminus was modified with a BHQ‐1 quencher. The strand hybridization and dissociation dynamics were analyzed by real‐time PCR fluorescence measurements to determine the melting temperature. The thermal analysis of the designed DNA system showed a melting temperature of approximately 31°C, as indicated by the peak of the derivative fluorescence trace in Figure 5b. Complete strand separation, crucial for effective payload release, was observed with fluorescence increase continuing up to approximately 36°C, which was also supported by independent SYBR Green I‐based DNA melting analysis (Figure S12).

To verify drug loading through nucleobase intercalation and quantify loading efficiency, fluorescence microscopy confirmed that doxorubicin (Dox) molecules were uniformly distributed across microflower surfaces (Figure 5c). Quantitative measurements demonstrated a maximum loading capacity of 10 µmol/g, representing 3 and 60‐fold improvements over DNA‐only and copper‐only carriers, respectively (Figure S13), attributed to the combined contributions of DNA intercalation and copper coordination [35]. When incorporated into thermosensitive PNIPAM hydrogels, the DNA‐Cu MF biohybrid hydrogel exhibited significantly lower drug leakage than control hydrogels under physiological conditions (Figure 5d; Figure S14). By evaluating drugs with diverse properties, we infer that the DNA‐Cu MF biohybrid hydrogel is governed by multiple competing interactions, including hydrophobic effects, Cu^2^^+^ coordination, and intermolecular aggregation, enabling efficient and broadly applicable drug loading (Table S2). Electro‐responsive release mechanism marks a substantial improvement over the conventional delivery approach. As shown in Figure 5e, applied voltages of 2–8 V generated predictable responses, with 8 V stimulation rapidly increasing local temperature to trigger drug release (∼3 µg/min) through combined DNA unfolding and hydrogel compression. On the other hand, the system shows inactive‐state stability with minimal background drug release (<0.5 µg/min) at subthreshold voltages (<3 V). In addition, quantitative analysis of volumetric shrinkage (Figure S15) demonstrated stable and repeatable actuation over 12 cycles, accompanied by consistent release performance under repeated electrical stimulation (5 V, 10 s on/1 min off, Figure 5f) and only minimal reduction in payload loading efficiency (less than 5.37 ± 1.68%, determined by fluorescence intensity analysis) after repeated activations. The hydrogel also exhibited pH‐responsive release behavior, with drug release at pH 5.0 being 9‐fold higher than that at physiological pH 7.0 (Figure S16). Post‐stimulation SEM images further suggested a slightly more open and porous network, which may be associated with ion migration‐driven osmotic swelling and partial reduction in coordination crosslinking density, while the overall morphology of the DNA‐Cu microflowers remained largely preserved (Figure S17) [36].

To validate the capacity for on‐demand and stepwise payload release, the biohybrid system was subjected to varying electrical parameters. Figure 5g demonstrates that the fluorescence intensity of the released drug rises steadily with escalating applied voltages. Furthermore, Figure S18 illustrates the release kinetics under a constant 8 V stimulation, where the relative drug release amount follows a logarithmic growth pattern corresponding to the stimulation duration. These combined observations confirm that the precise dosage and release rate are highly tunable through the modulation of either stimulation voltage or exposure time. Such programmable activation effectively resolves the delayed response commonly observed in traditional electro‐responsive platforms, facilitating rapid and highly controlled therapeutic delivery. The localized electrothermal heating generated by the DNA‐copper microflowers precisely and rapidly triggers both molecular‐level DNA unfolding (Figure 5b) and macroscopic hydrogel contraction (Figure 4c), leading to efficient and tightly controlled on‐demand payload release. The combination of electrical stimulation, thermal responsiveness, and pH sensitivity allows for precisely controlled, multi‐stage payload release, offering new possibilities for next‐generation drug delivery systems.

### 2.4 Cellular‐Level Functional Validation of DNA‐Functionalized Biohybrid Systems

To assess the therapeutic potential of our biohybrid system, we conducted functional validation studies at the cellular level. Based on the DNA‐programmable design that enables tailored functionality across different biomedical applications through oligonucleotide sequence adjustments, we implemented this adaptable platform for two applications: tumor therapy using electrically controlled drug release and neural modulation through stimulus‐activated neurotransmitter delivery.

**FIGURE 6:**
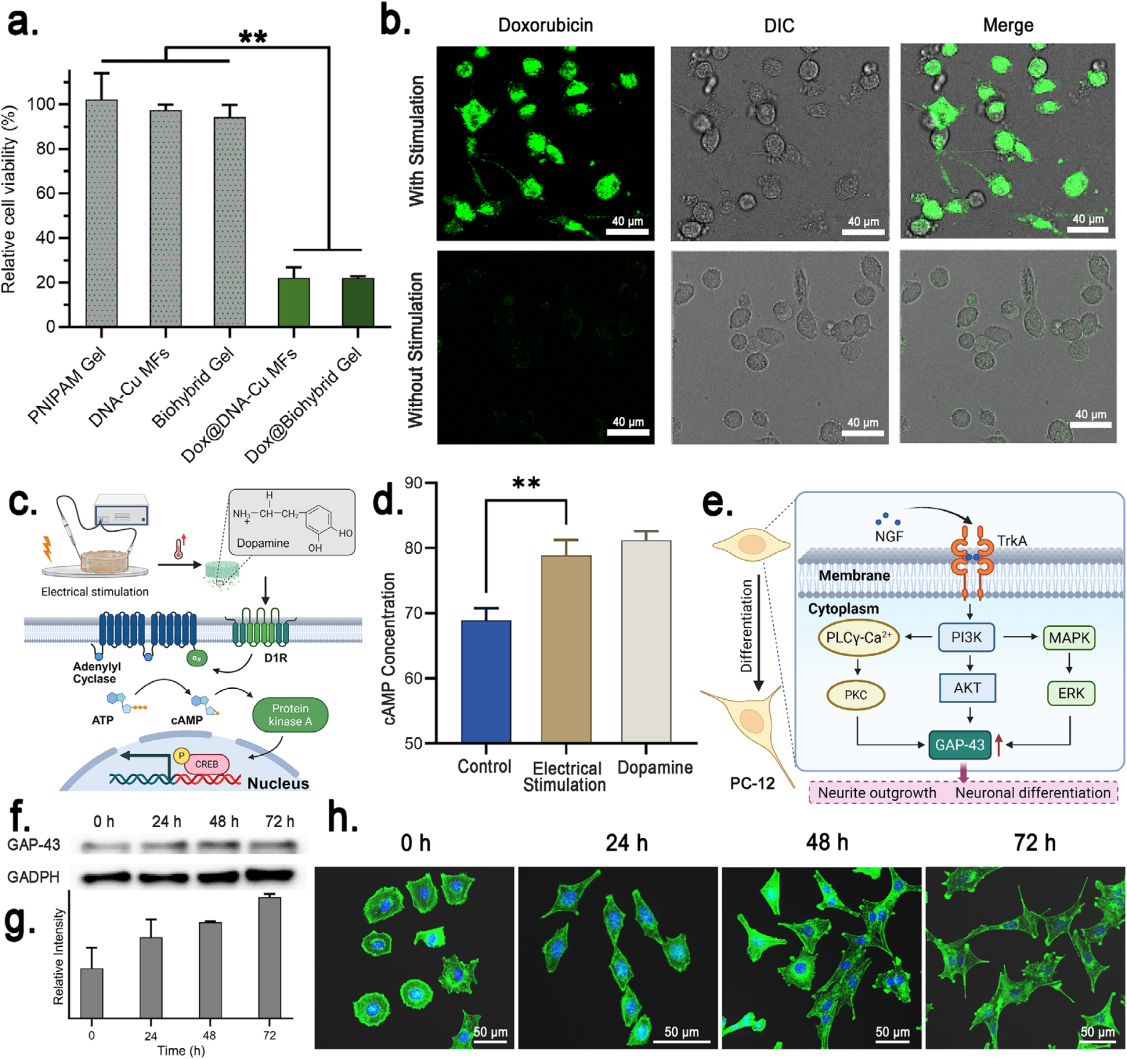
Validation of DNA‐functionalized biohybrid systems for tumor therapy and neuromodulation. (a) MDA‐MB‐231 cell viability measured by MTT assay following treatment with different formulations and electrical stimulation (8 V, 30 s) (n = 3, ^*^ *p* < 0.05, ^**^ *p* < 0.01). (b) Confocal microscopy images of intracellular doxorubicin distribution in MDA‐MB‐231 cells with (top) and without (bottom) electrical stimulation. (c) Schematic illustration of modular system reconstruction for neurotransmitter delivery through DNA sequence modification. (d) Intracellular cAMP levels in PC‐12 cells quantified by ELISA following dopamine release from biohybrid gels with/without electrical stimulation (8 V, 50 s). (e) Schematic illustration of growth factor‐induced PC‐12 cell differentiation signaling pathway. (f) Western blot analysis of GAP‐43 expression in PC‐12 cells during a 72 h differentiation period. (g) Relative GAP‐43 protein levels at 0, 24, 48, and 72 h were quantified based on band intensity analysis (n = 3, ^*^ *p* < 0.05, ^**^ *p* < 0.01). (h) Time‐lapse CLSM images documenting PC‐12 cell neurite outgrowth (0‐72 h) under differentiation conditions (scale bar: 50 µm).

We first evaluated the anticancer efficacy of our system using MDA‐MB‐231 breast cancer cells treated with multiple formulations, including Dox‐loaded DNA‐Cu MF hydrogel as the experimental group, along with conventional PNIPAM hydrogel, DNA‐Cu MFs alone, drug‐free Biohybrid Gels, and Dox‐loaded DNA‐Cu MFs as control groups. Under carefully controlled electrical stimulation conditions at 8 V for 30 s, quantitative analysis showed the Dox‐loaded biohybrid hydrogel achieved significantly better therapeutic outcomes with cell viability reduced to 22.00%, outperforming all control groups (*p* < 0.05, Figure 6a). This improved performance emerges from two complementary mechanisms where temperature‐sensitive DNA unfolding enables controlled drug release while simultaneous hydrogel contraction promotes active payload expulsion. Importantly, the drug‐free biohybrid hydrogel exhibited minimal cytotoxicity, confirming the material's biocompatibility for therapeutic use (Figure S19). Inductively coupled plasma (ICP) analysis revealed that the concentration of released Cu^2+^ in the medium was as low as 0.118 ± 0.02 ppm (∼1.86 µM), which is substantially below the reported thresholds affecting cellular physiological activity, further supporting the biosafety of the system [37, 38]. Confocal microscopy further verified the system's stimulus‐responsive properties, revealing three times greater cellular uptake of doxorubicin following electrical stimulation compared to unstimulated controls (Figure 6b), which validates both the triggered release capability and effective intracellular drug delivery.

To further prove the system's design flexibility for neural engineering applications, we engineered a neurotransmitter delivery platform by reprogramming the DNA sequences from the anticancer configuration to incorporate dopamine‐specific aptamers. Dopamine, as a crucial [39, 40], was effectively loaded and released by the reconfigured microflowers. Using PC‐12 neuronal cells, we demonstrated that electrical stimulation (8 V, 50 s) triggered dopamine‐mediated physiological responses characteristic of neural differentiation, which represents a fundamental process in stem cell therapy for treating neurological disorders (Figure 6c). As shown in Figure 6d, quantitative ELISA showed that cAMP levels increased from 68.957 pmol/mL in the non‐stimulated control to 78.868 pmol/mL upon electrical stimulation of the dopamine‐loaded DNA‐Cu MF hydrogel, consistent with the canonical dopamine‐D1 receptor signaling pathway essential for neuroplasticity [41, 42]. Further supporting these findings, PC‐12 cells cultured with nerve growth factor‐loaded hydrogels exhibited characteristic neuronal differentiation markers following periodic electrical stimulation (Figure 6e). Western blot analysis showed progressive upregulation of GAP‐43, a crucial regulator of axonal growth and synaptic plasticity [43, 44], with expression levels peaking at 72 h (Figure 6f,g). This molecular change paralleled the morphological evolution from rounded cells to neurite‐extending phenotypes observable by optical microscopy (Figure 6h). Our findings establish this system as a multifunctional platform that effectively delivers tumor drugs while simultaneously supporting neural regeneration, with its modular DNA‐based design enabling adaptable therapeutic applications.

### 2.5 Animal‐Level Therapeutic Validation of DNA‐Functionalized Biohybrid Systems

**FIGURE 7:**
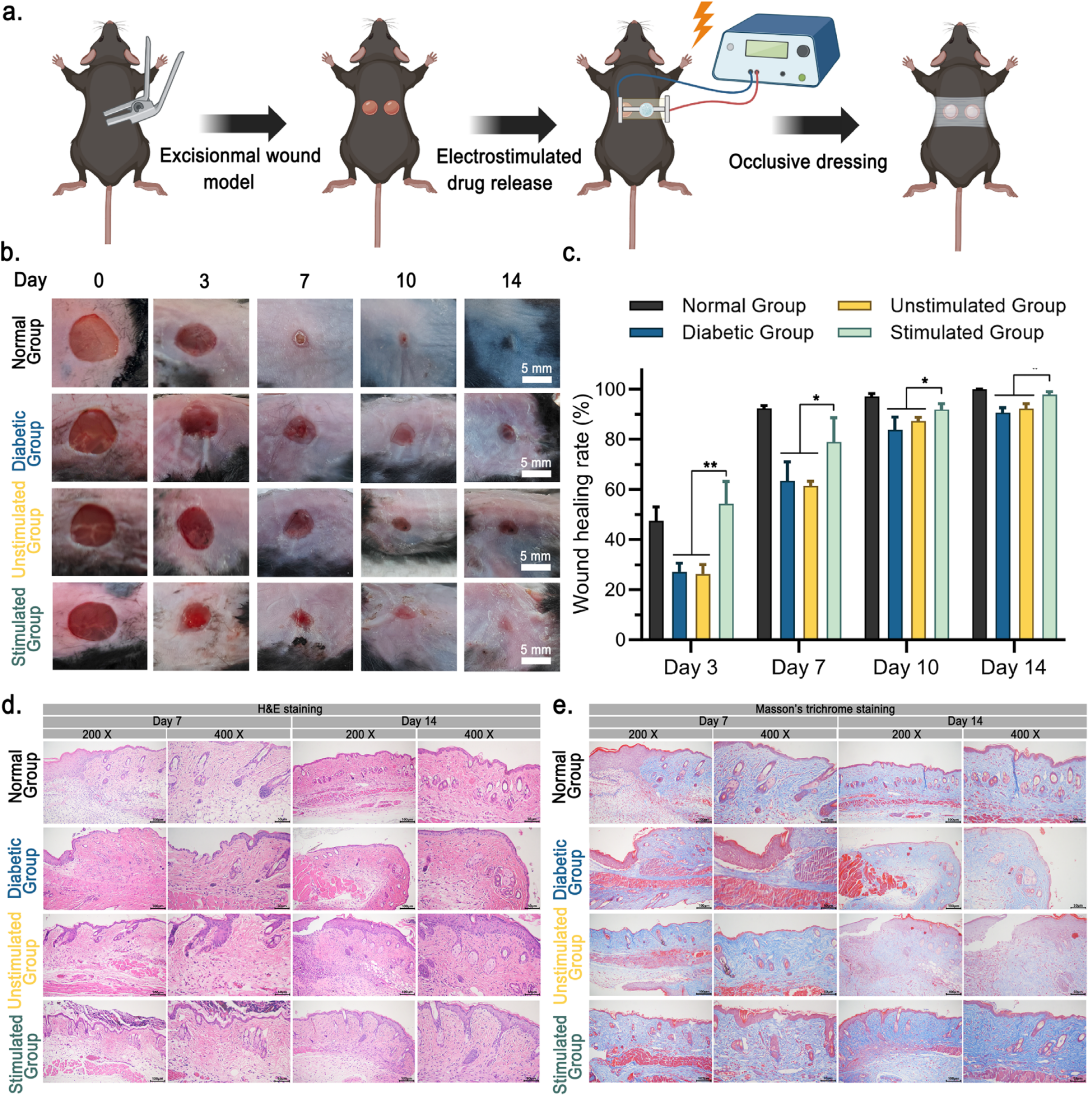
In vivo evaluation of DNA‐functionalized biohybrid system for diabetic wound healing. (a) Experimental setup showing the electrical stimulation method for triggered drug release. (b) Representative macroscopic images of wound healing progression for treatment groups at days 0, 3, 7, 10, and 14 post‐injury (scale bar: 5 mm). (c) Quantitative analysis of wound closure rates comparing normal controls, diabetic controls, non‐stimulated hydrogel group, and electrically stimulated hydrogel group (n = 3, ^*^ *p* < 0.05, ^**^ *p* < 0.01). (d,e) Histological assessment using H&E (d) and Masson (e) staining, revealing wound tissue morphology and collagen deposition at days 7 and 14 (scale bars: 100 µm at 200×, 50 µm at 400×).

Chronic wounds represent a major clinical challenge in diabetic patients, with impaired healing resulting from persistent inflammation and compromised tissue regeneration [45, 46, 47]. To address this critical medical need, we evaluated the therapeutic efficacy of our DNA‐integrated biohybrid system using streptozotocin‐induced diabetic C57BL/6J mice housed under specific pathogen‐free conditions. A custom device with two gold‐coated electrodes separated by a 2 mm gap was used. The hydrogel was placed between the electrodes, allowing the released solution to flow out while retaining the hydrogel. Following creation of full‐thickness dorsal wounds (8 mm diameter), animals were randomly assigned to four treatment groups: normal controls, diabetic controls, hydrogel‐treated without stimulation, and hydrogel‐treated with electrical stimulation (±8 V, 30 s, pulse width 0.1 s) (Figure 7a). The application of 8 V in short, pulsed electrical stimulation for localized delivery aligns with established biomedical interfaces for therapies such as electroporation and neuromodulation, where precise control of pulse duration and intensity is paramount [48, 49, 50]. The insulin‐loaded biohybrid hydrogel was carefully applied to wound surfaces and secured with sterile dressings, with electrical stimulation administered immediately prior to dressing application on treatment days. Longitudinal monitoring revealed significantly accelerated wound closure in the stimulated group, achieving 54.22 ± 8.73% healing by day 3 compared to 27.16 ± 2.67% in diabetic controls (*p* < 0.01). Complete re‐epithelialization (97.87 ± 1.12%) was observed by day 14, comparable to normal wound healing and markedly improved over unstimulated diabetic wounds (Figure 7b,c).

Histopathological evaluation provided evidence of enhanced tissue regeneration. At day 7, H&E staining demonstrated significantly reduced inflammatory cell infiltration in electrically stimulated wounds compared to diabetic controls, along with early epidermal regeneration and capillary formation comparable to normal healing. By day 14, complete epidermal restoration with mature appendages was observed in stimulated wounds, contrasting with persistent inflammation and incomplete healing in non‐stimulated diabetic wounds (Figure 7d). Masson's trichrome staining corroborated these findings, revealing well‐organized, dense collagen deposition in stimulated wounds that closely resembled normal tissue architecture, while untreated diabetic wounds exhibited sparse, disorganized collagen fibers (Figure 7e). Moreover, systemic analysis of major organs confirmed the treatment's safety profile, with no observed structural or functional alterations in heart, liver, spleen, lung, or kidney tissues harvested at day 14 (Figure S20). These findings demonstrate that the DNA‐integrated biohybrid system achieves accelerated diabetic wound healing through electrically controlled antibiotic release, effectively restoring the physiological healing process by regulating inflammation, angiogenesis, and collagen remodeling in diabetic conditions.

## 3 Conclusion

In this study, we present a biohybrid system that integrates DNA nanotechnology with electroresponsive hydrogels to achieve precise molecular delivery. By coupling electrically triggered heating with simultaneous DNA conformational changes and hydrogel contraction, the biohybrid gel overcomes challenges in controlled release systems regarding both precision and biocompatibility. Cellular studies demonstrate successful applications ranging from tumor therapy to neural modulation, while animal experiments confirm therapeutic efficacy in diabetic wound healing. The DNA‐programmable nature of this system allows straightforward adaptation to diverse biomedical needs through sequence modifications, establishing a versatile platform with significant potential for precision medicine applications, including oncology, neurotechnology, and regenerative medicine.

## Conflicts of Interest

None of the authors has a conflicts of interest to disclose

## Supporting information




**Supporting File 1**: advs75573‐sup‐0001‐SuppMat.docx.

**Supporting File 2**: advs75573‐sup‐0002‐MovieS1.docx.

## Data Availability

The data that support the findings of this study are available in the supplementary material of this article.

## References

[1] A. H. Khalbas , T. M. Albayati , N. S. Ali , and I. K. Salih , “Drug Loading Methods and Kinetic Release Models Using of Mesoporous Silica Nanoparticles as a Drug Delivery System: A Review,” South African Journal of Chemical Engineering 50, no. 1 (2024): 261–280, 10.1016/j.sajce.2024.08.013.

[2] L. Chen , R. Nixon , and G. De Bo , “Force‐Controlled Release of Small Molecules With a Rotaxane Actuator,” Nature 628, no. 8007 (2024): 320–325, 10.1038/s41586-024-07154-0. 38600268 PMC11006608

[3] S. Safaeian Laein , I. Katouzian , M. R. Mozafari , et al., “Biological and Thermodynamic Stabilization of Lipid‐Based Delivery Systems Through Natural Biopolymers; Controlled Release and Molecular Dynamics Simulations,” Critical Reviews in Food Science and Nutrition 64, no. 22 (2024): 7728–7747, 10.1080/10408398.2023.2191281. 36950963

[4] X. Hu , C. Zhang , Y. Xiong , S. Ma , C. Sun , and W. Xu , “A Review of Recent Advances in Drug Loading, Mathematical Modeling and Applications of Hydrogel Drug Delivery Systems,” Journal of Materials Science 59, no. 32 (2024): 15077–15116, 10.1007/s10853-024-10103-x.

[5] M. Sun , R. Song , Y. Fang , J. Xu , Z. Yang , and H. Zhang , “DNA‐Based Complexes and Composites: A Review of Fabrication Methods, Properties, and Applications,” ACS Applied Materials & Interfaces 16, no. 39 (2024): 51899–51915, 10.1021/acsami.4c13357. 39314016

[6] Y. Tian , M. Sun , R. Song , Z. Yang , and H. Zhang , “DNA Dendrimer‐Based Nanocarriers for Targeted Co‐delivery and Controlled Release of Multiple Chemotherapeutic Drugs,” RSC Advances 15, no. 4 (2025): 2981–2987, 10.1039/D4RA07839J. 39882009 PMC11775498

[7] Y. Wei , J. Lv , S. Zhu , S. Wang , J. Su , and C. Xu , “Enzyme‐Responsive Liposomes for Controlled Drug Release,” Drug Discovery Today 29, no. 7 (2024): 104014, 10.1016/j.drudis.2024.104014. 38705509

[8] Y. Ma , M. Yu , Z. Sun , et al., “Biomass‐Based, Dual Enzyme‐Responsive Nanopesticides: Eco‐Friendly and Efficient Control of Pine Wood Nematode Disease,” Acs Nano 18, no. 21 (2024): 13781–13793, 10.1021/acsnano.4c02031. 38752333

[9] Y. Yang , K. Long , Y. Chu , H. Lu , W. Wang , and C. Zhan , “Photoresponsive Drug Delivery Systems: Challenges and Progress,” Advanced Functional Materials 34, no. 38 (2024): 2402975, 10.1002/adfm.202402975.

[10] A. M. Agiba , N. Elsayyad , H. N. ElShagea , et al., “Advances in Light‐Responsive Smart Multifunctional Nanofibers: Implications for Targeted Drug Delivery and Cancer Therapy,” Pharmaceutics 16, no. 8 (2024): 1017, 10.3390/pharmaceutics16081017. 39204362 PMC11359459

[11] Y. Huang , K. Yao , Q. Zhang , et al., “Bioelectronics for Electrical Stimulation: Materials, Devices and Biomedical Applications,” Chemical Society Reviews 53 (2024): 8632–8712. 39132912 10.1039/d4cs00413b

[12] R. Bao , S. Wang , X. Liu , et al., “Neuromorphic Electro‐Stimulation Based on Atomically Thin Semiconductor for Damage‐Free Inflammation Inhibition,” Nature Communications 15, no. 1 (2024): 1327, 10.1038/s41467-024-45590-8. PMC10864345 38351088

[13] L. Yang , Y. Zhang , W. Cai , et al., “Electrochemically‐Driven Actuators: From Materials to Mechanisms and From Performance to Applications,” Chemical Society Reviews 53, no. 11 (2024): 5956–6010, 10.1039/D3CS00906H. 38721851

[14] A. Perez‐Nava , J. B. Gonzalez‐Campos , and F.‐U. BA , “Conducting Polymers for In Situ Drug Release Triggered via Electrical Stimulus,” ACS Applied Polymer Materials 6, no. 16 (2024): 9375–9395, 10.1021/acsapm.4c01013.

[15] M. E. Alkahtani , M. Elbadawi , C. A. Chapman , et al., “Electroactive Polymers for On‐Demand Drug Release,” Advanced healthcare materials 13, no. 3 (2024): 2301759. 37861058 10.1002/adhm.202301759 PMC11469020

[16] W. Duan , U. A. Robles , L. Poole‐Warren , and D. Esrafilzadeh , “Bioelectronic Neural Interfaces: Improving Neuromodulation Through Organic Conductive Coatings,” Advanced Science 11, no. 27 (2024): 2306275, 10.1002/advs.202306275. 38115740 PMC11251570

[17] E. Kim , S. Kim , Y. W. Kwon , et al., “Electrical Stimulation for Therapeutic Approach,” Interdisciplinary Medicine 1, no. 2 (2023): 20230003, 10.1002/INMD.20230003.

[18] A. Mittal , M. Ierapetritou , and D. G. Vlachos , “Joule‐Heating Materials for Advanced Chemical Manufacturing,” Chemical Engineering Journal 520 (2025): 166348, 10.1016/j.cej.2025.166348.

[19] H. Guo , L. Lu , F. L. Hatton , et al., “Wearable Body Temperature Sensing With Autonomous Self‐Regulated Joule Heating and Passive Cooling for Healthcare Applications,” Advanced Functional Materials 35, no. 13 (2025): 2417961, 10.1002/adfm.202417961.

[20] X.‐Q. Zheng , J.‐F. Huang , J.‐L. Lin , et al., “Controlled Release of Baricitinib From a Thermos‐Responsive Hydrogel System Inhibits Inflammation by Suppressing JAK2/STAT3 Pathway in Acute Spinal Cord Injury,” Colloids and Surfaces B: Biointerfaces 199 (2021): 111532, 10.1016/j.colsurfb.2020.111532. 33385822

[21] S. Chatterjee and P. C. Hui , “Review of Applications and Future Prospects of Stimuli‐Responsive Hydrogel Based on Thermo‐Responsive Biopolymers in Drug Delivery Systems,” Polymers 13, no. 13 (2021): 2086, 10.3390/polym13132086. 34202828 PMC8272167

[22] K. Ryu , G. Li , K. Zhang , J. Guan , Y. Long , and Z. Dong , “Thermoresponsive Hydrogels for the Construction of Smart Windows, Sensors, and Actuators,” Accounts of Materials Research 6, no. 3 (2025): 379–392, 10.1021/accountsmr.5c00007.

[23] A. Chakraborty , S. Alexander , W. Luo , et al., “Engineering Multifunctional Adhesive Hydrogel Patches for Biomedical Applications,” Interdisciplinary Medicine 1, no. 4 (2023): 20230008, 10.1002/INMD.20230008.

[24] D. Park , S. J. Lee , and J.‐W. Park , “Aptamer‐Based Smart Targeting and Spatial Trigger–Response Drug‐Delivery Systems for Anticancer Therapy,” Biomedicines 12, no. 1 (2024): 187, 10.3390/biomedicines12010187. 38255292 PMC10813750

[25] H. Zhang , Y. Pan , Y. Hou , et al., “Smart Physical‐Based Transdermal Drug Delivery System:Towards Intelligence and Controlled Release,” Small 20, no. 9 (2024): 2306944, 10.1002/smll.202306944. 37852939

[26] Z. Liao , T. Liu , Z. Yao , T. Hu , X. Ji , and B. Yao , “Harnessing Stimuli‐Responsive Biomaterials For Advanced Biomedical Applications,” Exploration 5 (2025): 20230133. 40040822 10.1002/EXP.20230133 PMC11875454

[27] P. Halvaeikhanekahdani , S. Zandi , Q. Ahmad , and H. Payravand , “Nanoparticle‐Integrated Electrostimulation‐Responsive Biomaterials: Innovations In Drug Delivery And Tissue Repair In Clinical Regenerative Medicine,” Interdisciplinary Medicine 4 (2026): 70077.

[28] G. Song , D. Chen , X. Zhang , et al., “Direct and Ultrafast Preparation of Cu_3_ (PO_4_)_2_ Nanoflower by Ultrasonic Spray Method Without Protein Assistant and Its Applications: Large‐scale Simulation and Catalytic Reduction,” Journal of Molecular Liquids 328 (2021): 115348, 10.1016/j.molliq.2021.115348.

[29] Z. Wang , J. Tu , P. Dong , Y. Bai , J. Han , and G. Xie , “BSA‐Cu_3_ (PO_4_)_2_ Hybrid Nanoflowers as a High‐Performance Redox Indicator for Robust Label‐Free Electrochemical Immunoassay,” Analytica Chimica Acta 1210 (2022): 339873, 10.1016/j.aca.2022.339873. 35595359

[30] P. Ma , R. Bi , Q. Wang , L. Lu , X. Ma , and F. Chen , “In Situ Synthesis Copper Phosphate‐protein Hybrid Nanoflower on Nickel Foam for the Sensitive Detection of Glucose in Body Fluids,” Materials Research Bulletin 170 (2024): 112583, 10.1016/j.materresbull.2023.112583.

[31] H. Fattahimoghaddam , I. H. Kim , K. Dhandapani , Y. J. Jeong , and A. TK , “Copper‐Nanoparticle‐Decorated Hydrothermal Carbonaceous Carbon–Polydimethylsiloxane Nanocomposites: Unveiling Potential in Simultaneous Light‐Driven Interfacial Water Evaporation and Power Generation,” Small 20, no. 37 (2024): 2403565, 10.1002/smll.202403565. 38738743

[32] O. C. Güven , M. Kar , and F. D. Koca , “Synthesis of Cherry Stalk Extract Based Organic@ Inorganic Hybrid Nanoflowers as a Novel Fenton Reagent: Evaluation of Their Antioxidant, Catalytic, and Antimicrobial Activities,” Journal of Inorganic and Organometallic Polymers and Materials 32, no. 3 (2022): 1026–1032.

[33] E. Taillandier and J. Liquier , “Infrared Spectroscopy of DNA,” Methods in enzymology 211 (1992): 307–335. 1406313 10.1016/0076-6879(92)11018-e

[34] F. Crisafuli , E. Ramos , and M. Rocha , “Characterizing the Interaction Between DNA and GelRed Fluorescent Stain,” European Biophysics Journal 44, no. 1 (2015): 1–7, 10.1007/s00249-014-0995-4. 25391339

[35] C. Pérez‐Arnaiz , N. Busto , J. M. Leal , and B. García , “New Insights Into the Mechanism of the DNA/Doxorubicin Interaction,” The Journal of Physical Chemistry B 118, no. 5 (2014): 1288–1295, 10.1021/jp411429g. 24417409

[36] T. Wallmersperger and D. Ballhause , “Coupled Chemo‐Electro‐Mechanical Finite Element Simulation Of Hydrogels: II Electrical Stimulation,” Smart Materials and Structures 17, no. 4 (2008): 045012.

[37] L. Fowler and H. Engqvist , “Effect of Copper Ion Concentration on Bacteria and Cells,” Materials 12, no. 22 (2019): 3798, 10.3390/ma12223798. 31752323 PMC6888263

[38] C. Grillo , M. Reigosa , and M. F. L. de Mele , “Effects of Copper Ions Released From Metallic Copper on CHO‐K1 Cells,” Mutation Research/Genetic Toxicology and Environmental Mutagenesis 672, no. 1 (2009): 45–50, 10.1016/j.mrgentox.2008.09.012. 18952000

[39] L. Speranza , U. Di Porzio , D. Viggiano , A. de Donato , and F. Volpicelli , “Dopamine: The Neuromodulator of Long‐term Synaptic Plasticity, Reward and Movement Control,” Cells 10, no. 4 (2021): 735, 10.3390/cells10040735. 33810328 PMC8066851

[40] Y. Cai , L. Xing , T. Yang , et al., “The Neurodevelopmental Role of Dopaminergic Signaling in Neurological Disorders,” Neuroscience Letters 741 (2021): 135540, 10.1016/j.neulet.2020.135540. 33278505

[41] M.‐O. Frégeau , M. Carrier , and G. Guillemette , “Mechanism of Dopamine D2 Receptor‐Induced Ca2+ Release in PC‐12 Cells,” Cellular Signalling 25, no. 12 (2013): 2871–2877. 24055909 10.1016/j.cellsig.2013.08.021

[42] F. Bono , Z. Tomasoni , V. Mutti , et al., “G Protein‐Dependent Activation of the PKA‐Erk1/2 Pathway by the Striatal Dopamine D1/D3 Receptor Heteromer Involves Beta‐Arrestin and the Tyrosine Phosphatase Shp‐2,” Biomolecules 13, no. 3 (2023): 473, 10.3390/biom13030473. 36979407 PMC10046256

[43] L. do Amaral , N. A. G. Dos Santos , F. M. Sisti , and E. Del Bel , “The antibiotic doxycycline mimics the NGF signaling in PC12 Cells: A Relevant Mechanism for Neuroprotection,” Chemico‐Biological Interactions 341 (2021): 109454, 10.1016/j.cbi.2021.109454. 33798505

[44] L. Amaral , G. R. Caldas , N. A. G. D. Santos . R. L. T. Parreira , J. K. Bastos , and A. C. D. Santos , “Baccharin From Brazilian Green Propolis Induces Neurotrophic Signaling Pathways in PC12 Cells: Potential for Axonal and Synaptic Regeneration,” Naunyn‐Schmiedeberg's Archives of Pharmacology 395, no. 6 (2022): 659–672. 35246694 10.1007/s00210-022-02224-4

[45] V. Falanga , R. R. Isseroff , A. M. Soulika , et al., “Chronic Wounds,” Nature Reviews Disease Primers 8, no. 1 (2022): 50, 10.1038/s41572-022-00377-3. PMC10352385 35864102

[46] N. Rodríguez‐Rodríguez , I. Martínez‐Jiménez , A. García‐Ojalvo , et al., “Wound Chronicity, Impaired Immunity and Infection in Diabetic Patients,” MEDICC Review 24 (2022): 44–58, 10.37757/MR2021.V23.N3.8. 34653116

[47] J. L. Burgess , W. A. Wyant , B. A. Abujamra , R. S. Kirsner , and I. Jozic , “Diabetic Wound‐Healing Science,” Medicina 57, no. 10 (2021): 1072, 10.3390/medicina57101072. 34684109 PMC8539411

[48] Y. Ye , W. Zhao , J. Chen , et al., “Single‐Cell Electroporation and Real‐Time Electrical Monitoring on a Microfluidic Chip,” paper presented at: 2020 IEEE 33rd International Conference on Micro Electro Mechanical Systems (MEMS) (IEEE, 2020), 10.1109/MEMS46641.2020.9056217.

[49] N. Tazin , T. J. Stevenson , J. L. Bonkowsky , and B. K. Gale , “Using Electroporation to Improve and Accelerate Zebrafish Embryo Toxicity Testing,” Micromachines 15, no. 1 (2023): 49, 10.3390/mi15010049. 38258168 PMC10819337

[50] C.‐C. Hsieh and M.‐D. Ker , “Design of Multi‐Channel Monopolar Biphasic Stimulator for Implantable Biomedical Applications,” paper presented at: 2018 IEEE 61st International Midwest Symposium on Circuits and Systems (MWSCAS) (IEEE, 2018), 10.1109/MWSCAS.2018.8623995.

