# Supplementary material for: Versatile DNA‐Functionalized Biohybrid Hydrogel Platforms for Electrothermally Activated On‐Demand Payload Release
Source: Adv Sci (Weinh). 2026 May 7;13(43):e75573. doi: 10.1002/advs.75573 (PMC13335934; doi:10.1002/advs.75573)
Supplement: Supplementary file 1 — Supporting File 1: advs75573‐sup‐0001‐SuppMat.docx. [file ADVS-13-e75573-s002.docx]

*Supporting Information for*

**Versatile DNA-Functionalized Biohybrid Hydrogel Platforms for Electrothermally Activated On-Demand Payload Release**

Mengqiu Sun^1,2^, Zhenshuai Tang^1^, Jia Zhang^1^, Rui Song^1^, Wei Luo^1^, Juan Huang^3^, Jincheng Zeng^3^, Zhen He^2,^*, Hao Zhang^1,3,^*

^1^ Guangdong Provincial Key Laboratory of Mathematical and Neural Dynamical Systems, Great Bay University, Dongguan, Guangdong 523000, China.

^2^ Department of Materials Science and Engineering, Southern University of Science and Technology, Shenzhen, Guangdong, 518055, China

^3^ Dongguan Key Laboratory of Medical Bioactive Molecular Developmental and Translational Research, Guangdong Provincial Key Laboratory of Medical Immunology and Molecular Diagnostics, Guangdong Medical University, Dongguan, 523808, China

* Correspondence: [zhanghao@gbu.edu.cn](mailto:zhanghao@gbu.edu.cn), [hez@sustech.edu.cn](mailto:hez@sustech.edu.cn)

**1. Experimental Section**

**1.1 Materials**

Copper sheets were obtained from 3AMaterials. Sodium chloride (NaCl, AR grade) and phosphate buffer (0.1 M, pH 8.0) were sourced from Shanghai Yuanye Bio-Technology Co., Ltd. Chemical reagents including copper chloride (CuCl₂), magnesium chloride (MgCl₂), N,N'-methylenebisacrylamide (MBAM), N,N,N',N'-tetramethylethylenediamine (TEMED), and N-isopropylacrylamide were purchased from Adamas-beta. Doxorubicin hydrochloride (Dox, 99.57% purity) and paclitaxel (PTX, 98% purity) were acquired from Aladdin, Energy Chemical, and Macklin.

For biochemical assays, we obtained the following from Sangon Biotech (Shanghai): cAMP ELISA kit, BCA protein assay kit, Tris-HCl solution (1 M), non-fat powdered milk, high-sensitivity ECL luminescence reagent, anti-GAP43 primary antibody (rabbit polyclonal, Cat# D290563), HRP-conjugated goat anti-rabbit IgG secondary antibody (Cat# D110058), and anti-GAPDH antibody (rabbit polyclonal, Cat# D110016). Electrophoresis supplies including UNI™ PAGE running buffer, TBST buffer (10×), and PVDF transfer membranes were purchased from Affinibody LifeScience. GelRed nucleic acid stain was acquired from Shanghai Yuanye Bio-Technology Co., Ltd.

All DNA oligonucleotides were custom-synthesized by Sangon Biotech with HPLC purification (>95% purity). Detailed oligonucleotide sequences are provided in Table S1.

Table S1**.** DNA oligonucleotide sequences used in this study

| Name | Sequence (5' to 3') | Modification |
| --- | --- | --- |
| Y1 | CGCCGG T CCCGGC TATAAATAAATATAATAATT |  |
| Y2 | GCCGGG T GCGGGG TATAAATAAATATAATAATT |  |
| Y3 | CCCCGC T CCGGCG TATAAATAAATATAATAATT |  |
| S1 | AGTTTACTAAGAATG AATTATTATATTTATTTATA |  |
| S2 | CATTCTTAGTAAACT AATTATTATATTTATTTATA |  |
| SCY1 | AGTTTACTAAGAATG AATTATTATATTTATTTATA | 3’-BHQ-1 |
| SCY2 | CATTCTTAGTAAACT-AATTATTATATTTATTTATA | 3’-BHQ-1 |
| YCY1 | CGCCGG T CCCGGC-TATAAATAAATATAATAATT | 5’-FAM |

**1.2 Methods**

**Pre-treatment of copper sheets**

Circular copper sheets (purity ≥99.5%, thickness ~0.2 mm, diameter 5 mm) were fabricated using a stamping die and subsequently immersed in 0.1 M HCl solution for 1 min to remove the surface oxide layer. The treated copper sheets were then thoroughly rinsed with deionized water to eliminate residual acid prior to further use. The size of the copper precursor appears to affect the dimensions of the resulting copper nanoflowers. For instance, under identical experimental conditions, the use of copper powder (325 mesh, purity ≥99.5%) instead of copper sheets resulted in the formation of copper nanoflowers with reduced, nanoscale dimensions (Figure S21).

**Synthesis of DNA-Copper Microflowers**

A new synthetic approach was developed for fabricating DNA-copper microflowers. In this method, copper sheets were immersed in an aqueous solution containing DNA oligonucleotides, sodium chloride (0.3 M), and phosphate buffer (0.1 M, pH 8.0), followed by static incubation at room temperature for three days to allow the gradual formation of hierarchical microflower structures through copper ion coordination and DNA-directed crystallization.

**Drug Loading of DNA-Copper Microflowers**

The drug loading process was performed by incubating the as-synthesized DNA-copper microflowers with doxorubicin hydrochloride solution (4 mM in PBS, pH 7.4) under vigorous shaking for 5 minutes at room temperature. This efficient loading procedure enables the intercalation of doxorubicin molecules into the DNA components of the microflowers through both electrostatic interactions and intercalation binding.

**Synthesis of Thermosensitive Hydrogel**

The thermoresponsive PNIPAM-co-MBAM hydrogel was prepared according to an established protocol with modifications. Briefly, N-isopropylacrylamide monomer (1 g) was dissolved in ice-cold deionized water (9 mL, 2°C), followed by sequential addition of the crosslinker N,N'-methylenebisacrylamide (1 mg), initiator ammonium persulfate (10 mg), and catalyst N,N,N',N'-tetramethylethylenediamine (10 μL). The homogeneous mixture was allowed to polymerize for 1 hour at room temperature, resulting in the formation of a three-dimensional hydrogel network exhibiting temperature-responsive phase transition behavior.

**Electrode structure and hydrogel size**

Custom-designed platinum (Pt) or gold (Au) inert electrodes were used for electrical stimulation. The interelectrode distance was maintained at 1–3 mm to ensure sufficient current intensity while avoiding undesired drug accumulation near the electrode surface. Hydrogel samples were formed from 100 μL precursor solution in a 96-well plate to control sample height and ensure uniform current distribution. Excessive hydrogel thickness may hinder current propagation to the upper region, thereby affecting overall drug release.

**Drug Loading of Hydrogels**

Prior to drug loading, the hydrogels were pretreated by taking advantage of their thermo-responsive behavior. Specifically, the hydrogels were first heated to induce shrinkage and then cooled in PBS to allow reswelling, during which residual species within the gel matrix could be effectively exchanged and released. The samples were subsequently rinsed repeatedly with PBS (at least three times) to further remove residual unreacted NIPAM monomers and free copper microflowers, thereby reducing the potential toxicity of the hydrogel and improving the reliability of subsequent drug-loading experiments.

Doxorubicin-Loaded Hydrogel: The drug-loaded microflowers were uniformly dispersed in the precursor solution (NIPAM/MBAM) prior to the addition of the initiator system (APS/TEMED). After thorough mixing, polymerization was initiated, resulting in the formation of a doxorubicin-encapsulated hydrogel network with homogeneous drug distribution.

Dopamine-Loaded Hydrogel: Dopamine solution (10 μM) was prepared in 0.01 M HCl containing 0.1 μM ascorbic acid as an antioxidant. The microflower-loaded hydrogels underwent three cycles of thermal-triggered dehydration (40°C) and reswelling (room temperature PBS) to remove residual monomers and improve biocompatibility. The pretreated hydrogels were then equilibrated in dopamine solution to achieve complete loading before biological experiments.

Insulin-Loaded Hydrogel: Following the established pretreatment protocol, microflower-incorporated hydrogels were incubated with insulin solution (100 μg/mL) at room temperature until reaching absorption equilibrium. The insulin-loaded constructs were stored under appropriate conditions for subsequent therapeutic evaluation.

β-NGF-Loaded Hydrogel: Using the same biocompatibility-enhancing pretreatment, hydrogels were loaded with β-nerve growth factor (20 μg/mL) through equilibrium absorption at room temperature. The β-NGF-loaded hydrogels were preserved for following neural regeneration studies.

**Scanning Electron Microscopy (SEM)**

Hydrogel samples were cryo-fractured following immersion in liquid nitrogen at both 25 ℃ (ambient) and 45°C (above LCST) conditions. After freeze-drying for 24 hours to preserve microstructure, samples were sputter-coated and imaged using field emission SEM (JEOL JSM-6700F) operated at 1 kV accelerating voltage in secondary electron detection mode to characterize morphological changes associated with temperature-responsive phase transitions.

**Spectrophotometric Analysis**

Thermoresponsive optical properties were quantified using a temperature-controlled microplate reader (Varioskan Lux, Thermo Scientific). Hydrogel aliquots (100 μL) were dispensed in triplicate into 96-well plates and subjected to a controlled temperature ramp (25-40 ℃, 1°C increments) with 10-minute equilibration intervals. Absorbance measurements at 400 nm were acquired following each temperature stabilization period to construct temperature-dependent optical profiles.

**Confocal Laser Scanning Microscopy (CLSM)**

Microstructural characterization and drug distribution analysis were performed using a Leica Stellaris 8 confocal system. Samples containing fluorescently labeled components (Dox/GelRed-loaded microflowers) were imaged under appropriate excitation/emission parameters to visualize spatial organization and payload localization within the hydrogel matrix.

**Infrared Thermography**

Electrothermal response profiles were captured using a LUXET Thermo 50 lock-in infrared imaging system. Hydrogel specimens positioned between fork electrodes were subjected to 10 V applied potential while recording spatiotemporal temperature distributions at 50 ms resolution, enabling quantitative analysis of Joule heating characteristics and thermal transport properties.

**Inductively Coupled Plasma Optical Emission Spectrometry (ICP-OES)**

The concentration of copper ions in solution was quantified using a Thermo Scientific iCAP PRO X ICP-OES Duo. Calibration curves were established using a series of copper chloride standard solutions prepared in 5% (v/v) nitric acid. The sample solutions were subsequently analyzed under identical conditions, and the copper ion concentrations were determined by interpolation from the external calibration curve.

**Cell Culture**

MDA-MB-231 (human breast cancer) and B16 (murine melanoma) cell lines were maintained in Dulbecco's Modified Eagle Medium (DMEM) supplemented with 10% fetal bovine serum (FBS) and 1% penicillin-streptomycin antibiotic mixture. Cultures were incubated at 37 ℃ in a humidified 5% CO₂ atmosphere. For subculturing, confluent monolayers (>70% density) were detached using 0.25% trypsin-EDTA solution (3 min incubation), neutralized with complete medium, and passaged at 1:3 split ratios. Routine maintenance included daily medium replacement following PBS washing. For long-term preservation, cells were cryopreserved in freezing medium (10% DMSO, 10% FBS) using a controlled-rate freezing protocol (-20°C for 12 h followed by liquid nitrogen storage).

**Cytotoxicity Assessment**

The antiproliferative effects were evaluated using the MTT colorimetric assay. MDA-MB-231 cells were seeded in 96-well plates (5×10³ cells/well) and allowed to adhere overnight. Following treatment with test compounds (24-48 h), cells were incubated with MTT reagent (5 mg/mL in DMEM, 4 h at 37 ℃). The insoluble formazan product was solubilized in DMSO (2 h, dark incubation), and absorbance was quantified at 570 nm using a Varioskan Lux microplate reader (Thermo Scientific). Three independent experiments were performed with six replicates per condition.

**Quantification of cAMP**

Intracellular cAMP levels in PC-12 cells were measured using a commercial ELISA kit (Abcam ab65355) according to the manufacturer's protocol. Briefly, cell lysates were prepared and processed through the competitive immunoassay, with absorbance readings performed at 450 nm. cAMP concentrations were calculated against a standard curve generated with known concentrations of cAMP.

**Protein Expression Analysis**

Total cellular proteins were extracted using RIPA lysis buffer containing protease inhibitors. Protein samples (20 μg/lane) were resolved by 10% SDS-PAGE and electrotransferred to PVDF membranes. After blocking with 5% non-fat milk, membranes were probed with primary antibodies (overnight at 4°C) followed by HRP-conjugated secondary antibodies (1 h at room temperature). Protein bands were visualized using enhanced chemiluminescence (ECL) detection reagents on a ChemiDoc imaging system (Thermo Fisher Scientific). Band intensities were quantified using ImageJ software.

**Fluorescence Spectroscopy Analysis**

Fluorescence characterization of doxorubicin-loaded hydrogels was performed using an FL970 fluorescence spectrophotometer (Techcomp). Samples were excited at 495 nm with emission spectra collected from 520-700 nm at 1 nm resolution. Instrument parameters were standardized with excitation and emission slit widths fixed at 5 nm for all measurements. Spectra were baseline-corrected and normalized to account for potential scattering effects from the hydrogel matrix.

**Non-denaturing Polyacrylamide Gel Electrophoresis (PAGE)**

DNA strand integrity and hybridization efficiency were assessed by native PAGE analysis. A 10% polyacrylamide gel was prepared by mixing 1.0 g of 40% acrylamide/bis-acrylamide solution (29:1 ratio) with 1× TBE buffer, followed by addition of polymerization initiators (100 μL 10% APS and 10 μL TEMED). The solution was cast between glass plates with 1.0 mm spacers and allowed to polymerize for 30-45 minutes at room temperature. Following polymerization, the gel was mounted in a vertical electrophoresis system (Bio-Rad Mini-PROTEAN) containing 1× TBE running buffer. DNA samples (5 μL) were mixed with 6× loading buffer (1 μL) and loaded into individual wells. Electrophoresis was performed at constant voltage (220 V) for 40 minutes at room temperature. Post-electrophoresis, gels were stained with 1× GelRed nucleic acid stain for 15 minutes and imaged using a Bio-Rad GelDoc XR+ documentation system. Migration patterns were analyzed to verify strand purity, molecular weights, and hybridization efficiency of DNA constructs.

**Animal Experimental Protocol**

Animal Housing: Female C57BL/6J mice (8 weeks old) were obtained from Guangdong Provincial Experimental Animal Center and maintained under specific pathogen-free (SPF) conditions. Animals were housed in individually ventilated cages (29 × 14 × 13 cm) with controlled environmental parameters (22±1°C, 55±5% humidity, 12-hour light/dark cycle). All mice received ad libitum access to autoclaved food and water throughout the study period.

Diabetes Induction: Forty mice were stratified into four experimental groups using a randomized block design based on fasting blood glucose (FBG) levels (mean baseline: 5.32±0.42 mmol/L). Type 1 diabetes was induced via intraperitoneal injection of streptozotocin (STZ, 100 mg/kg/day in citrate buffer pH 4.5) for five consecutive days following a 5-hour fast. Control animals received vehicle injections. Diabetes confirmation was established one week post-induction (FBG >11.1 mmol/L; experimental group mean: 15.47±2.13 mmol/L).

Wound Healing Experiments: Full-thickness excisional wounds (8 mm diameter) were created on the dorsal surface under anesthesia. Treatment groups included: (1) Normal control (non-diabetic), (2) Diabetic control, (3) Hydrogel-treated diabetic (HG-off), and (4) Electrically stimulated hydrogel-treated diabetic (HG-on). HG-on group received ±10 V stimulation (30 s pulses, 0.1 s width) immediately before dressing application. Treatments were administered on days 0, 3, 7, 10, and 14 post-wounding.

Tissue Collection and Analysis: Wound tissues were harvested at predetermined intervals (days 0, 7, 14) for histological evaluation. Different magnification images were taken from adjacent sections, all obtained from the same wound of the same mouse, and exhibited comparable histological characteristics. Terminal collection included major organs (heart, liver, spleen, lung, kidney) for systemic safety assessment. All procedures were approved by the Institutional Animal Care and Use Committee and conducted in accordance with ARRIVE guidelines.

*All animal procedures were performed in accordance with the guidelines of the Animal Ethical and Welfare Committee (AEWC) of Shenzhen Glorybay Biotech Co.,Ltd., and approved under protocol number [No. RW-IACUC-25-0071].

**2. Supplementary Tables and Figures**


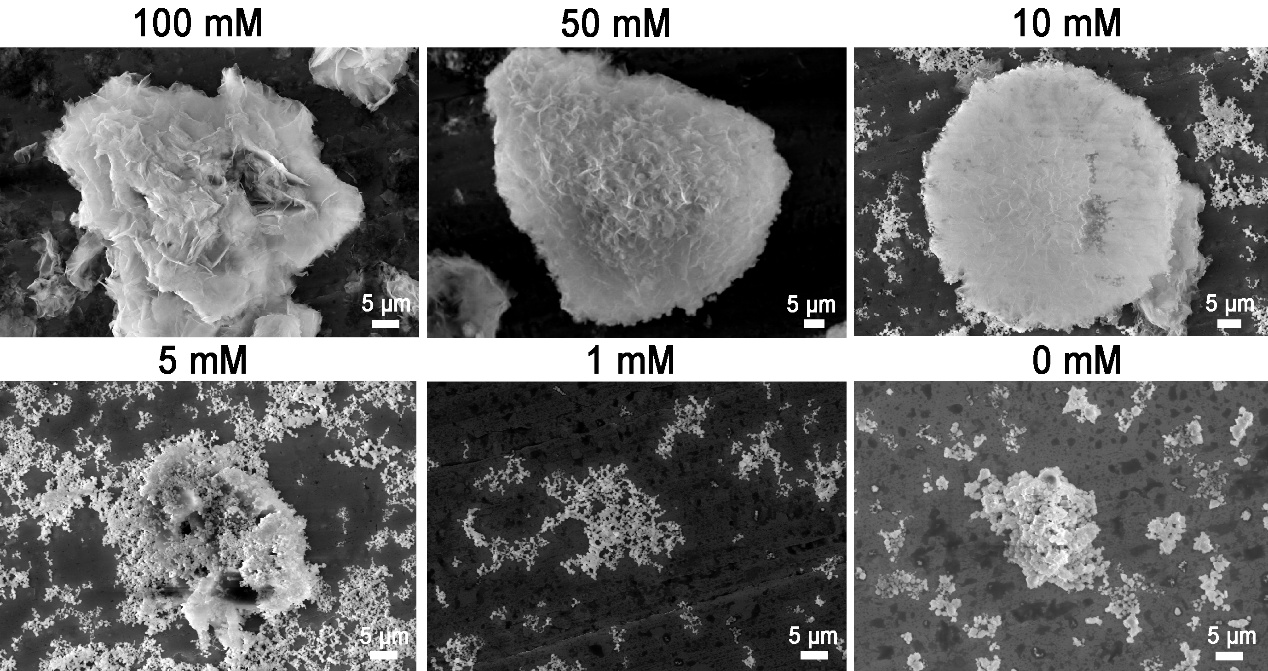


**Figure S1.** SEM images demonstrating morphological variations of copper microflowers synthesized at different PBS concentrations (0, 1, 5, 10, 50, and 100 mM). Scale bars: 5 μm.

**
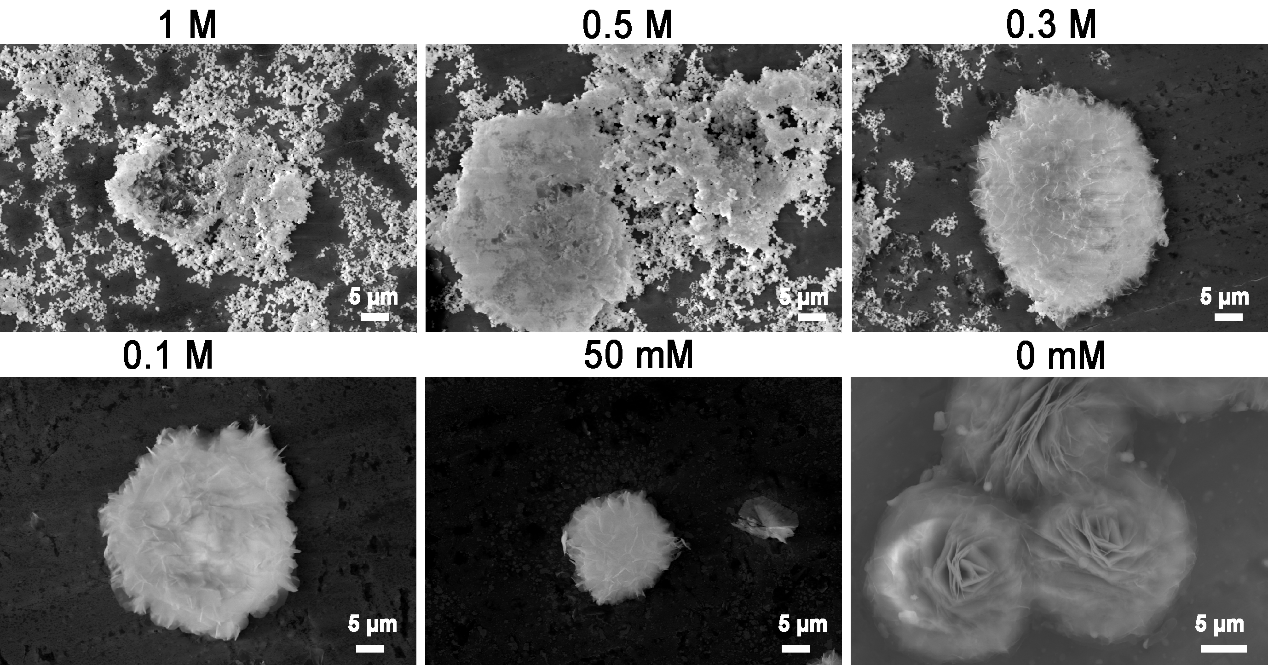
**

**Figure S2.** SEM analysis of copper microflowers prepared with varying NaCl concentrations (0, 0.05, 0.1, 0.3, 0.5, and 1 M). Scale bars: 5 μm.


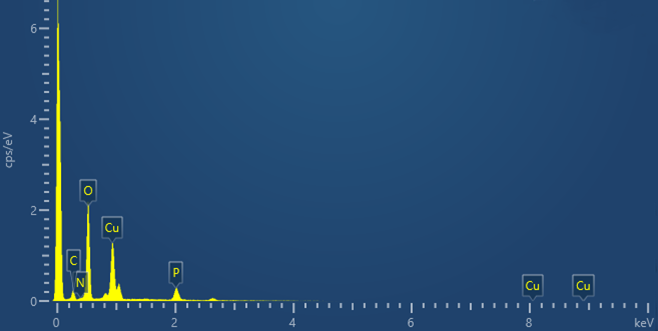


**Figure S3.** EDS elemental composition analysis of copper phosphate microflowers revealing Cu (40.61 wt%), P (17.66 wt%), O (35.90 wt%), and C (5.63 wt%) content.

**
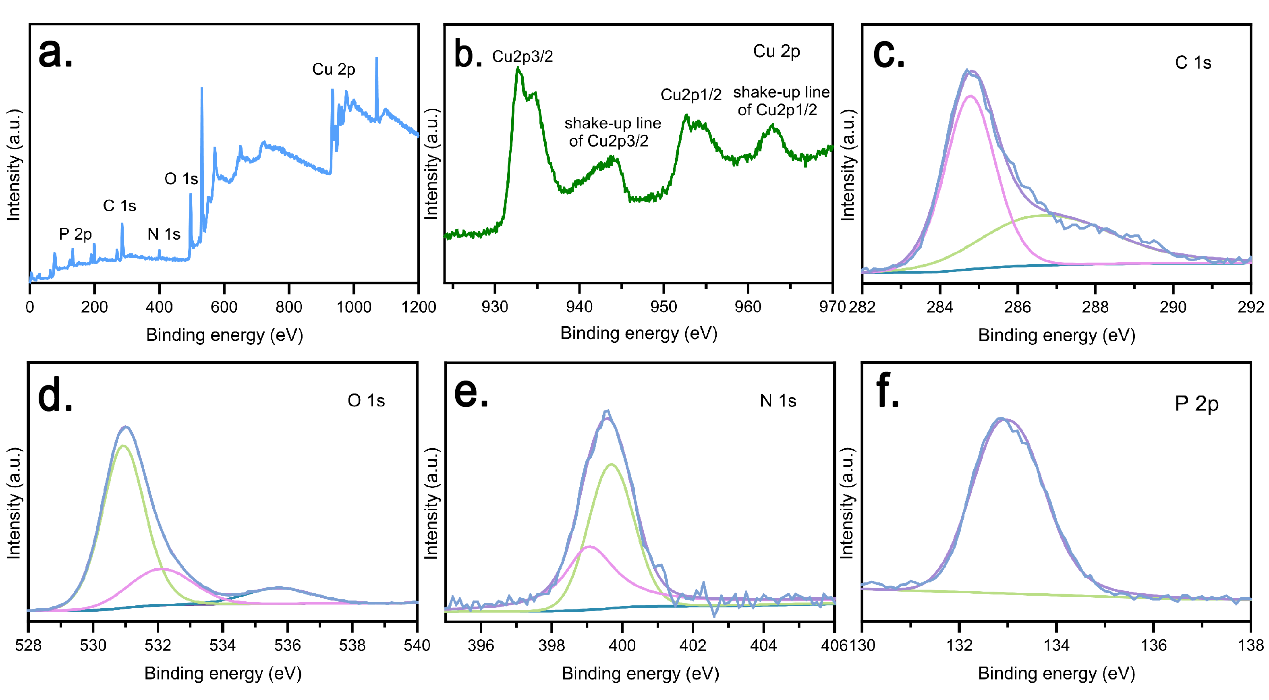
**

**Figure S4.** XPS characterization of copper microflowers: (a) Full survey spectrum; (b-f) High-resolution spectra of Cu 2p, C 1s, O 1s, N 1s, and P 2p orbitals.


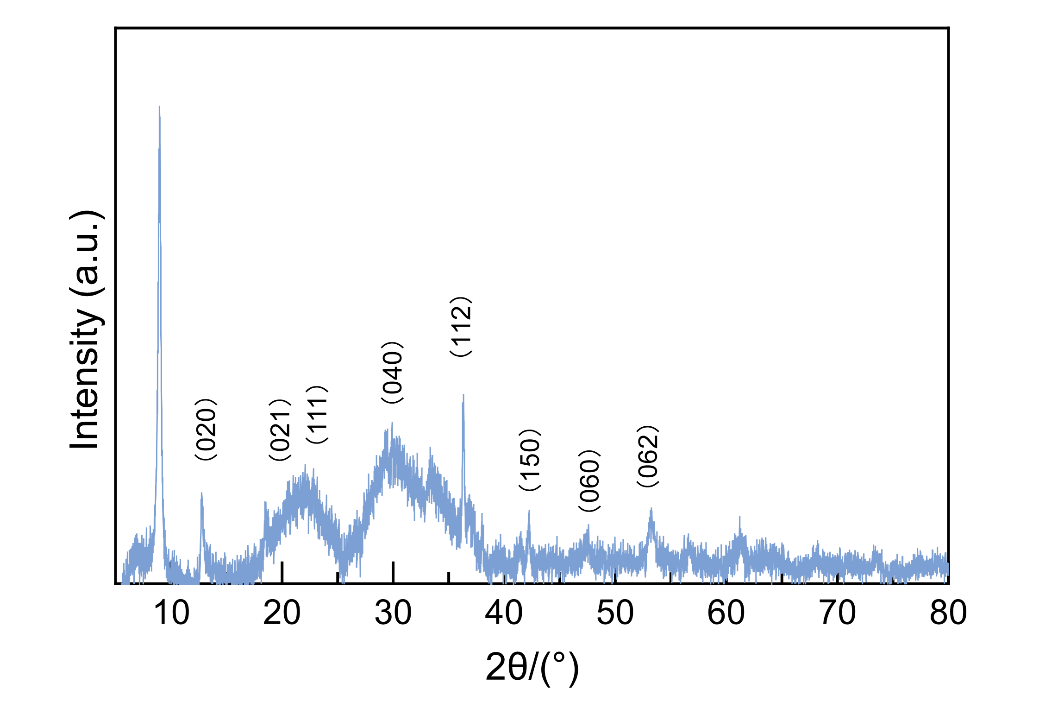


**Figure S5.** XRD diffraction patterns of DNA-functionalized microflowers.


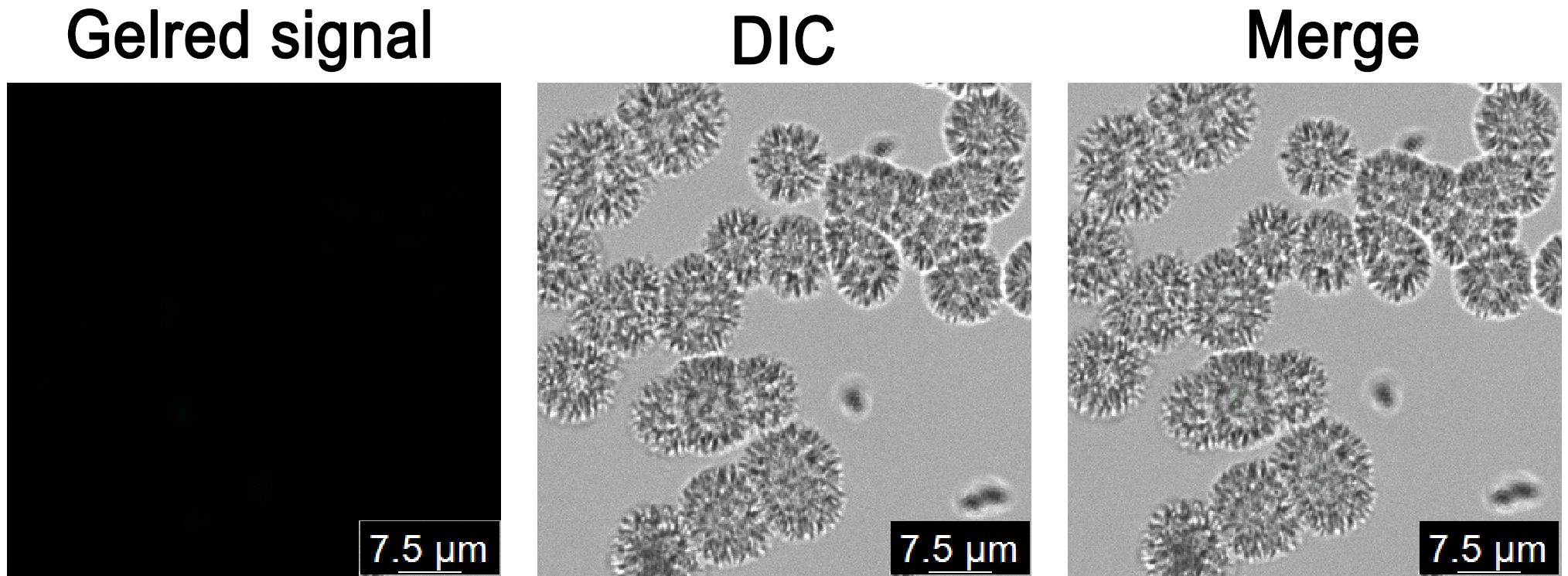


**Figure S6.** CLSM image of copper microflowers (without DNA) stained with GelRed nucleic acid dye (λ_ex_ = 518 nm). Scale bar: 7.5 μm.


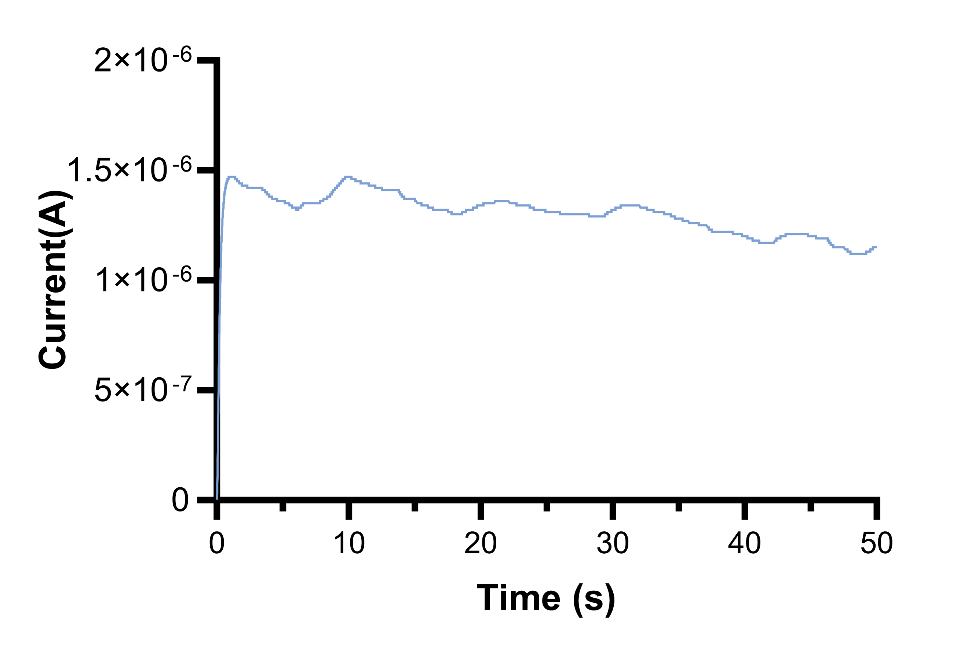


**Figure S7.** Electrical conductivity characterization of copper microflower powder. Current-time (I-t) curve recorded during application of a constant 5 V DC potential across the dried microflower sample.

**
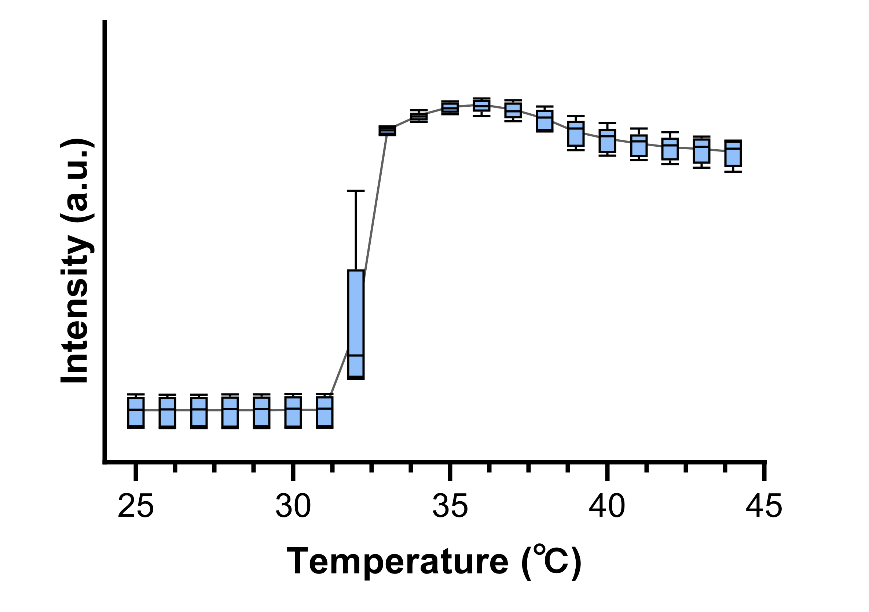
**

**Figure S8.** Temperature-dependent absorbance (400 nm) of thermosensitive hydrogel demonstrating phase transition behavior.


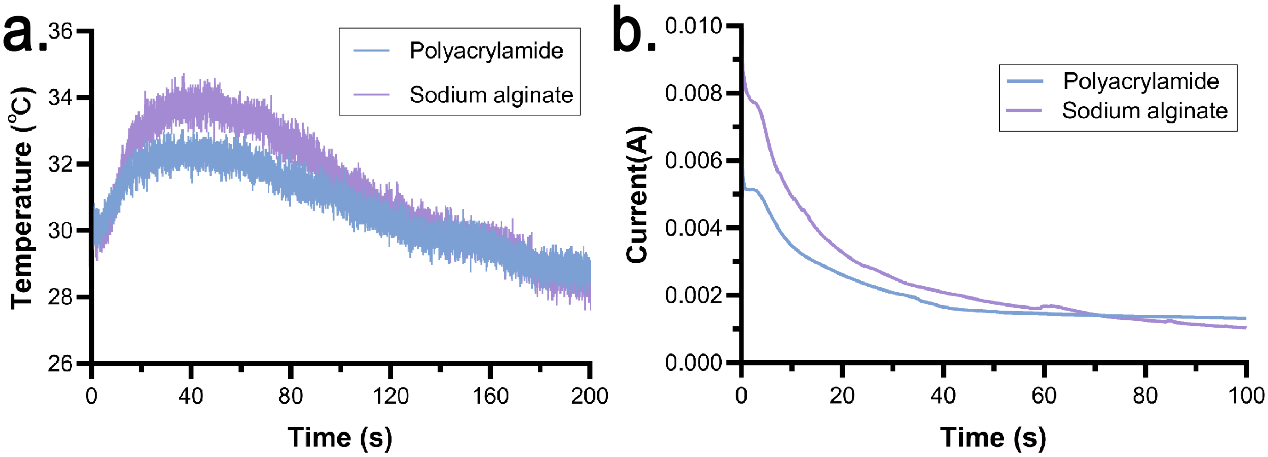


**Figure S9.** Comparative electrothermal response of control hydrogels: (a) Temperature-time curves of polyacrylamide hydrogel (blue) and sodium alginate hydrogel (purple) under 10 V DC stimulation; (b) Current-time characteristics of polyacrylamide hydrogel (blue) and sodium alginate hydrogel (purple) during electrical activation.

**
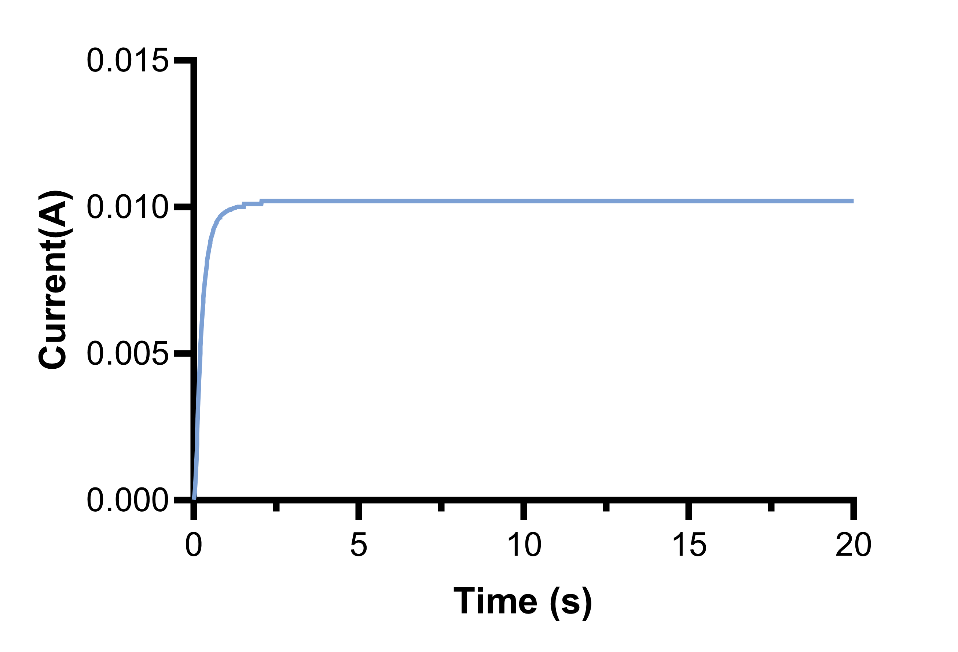
**

**Figure S10.** Current–time (i–t) curve of biohybrid hydrogels (2 mg microflowers/100 μL) under an applied voltage of 1 V for 20 s.


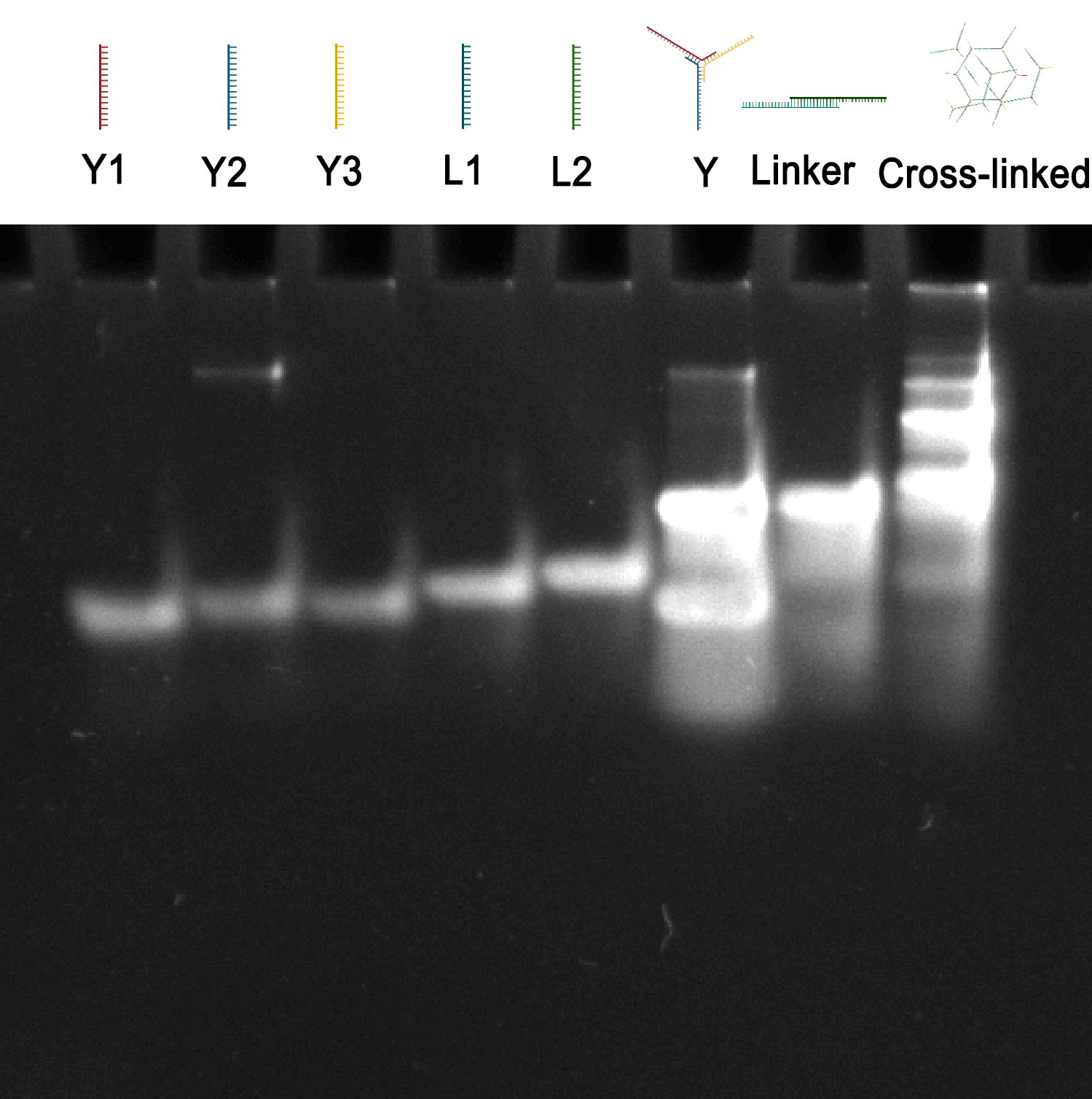


**Figure S11.** Native PAGE analysis of DNA constructs: Lane 1: Y1, Lane 2: Y2, Lane 3: Y3, Lane 4: L1, Lane 5: L2, Lane 6: Y-junction, Lane 7: Linker, Lane 8: Crosslinked product.

**
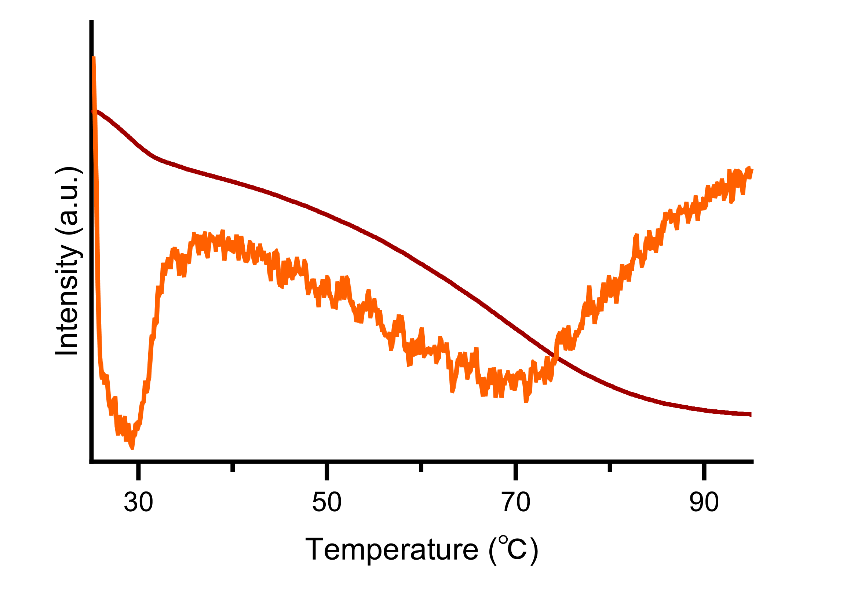
**

**Figure S12.** DNA melting temperature monitored by SYBR Green I fluorescence (red) with derivative plot (orange) indicating transition temperature.


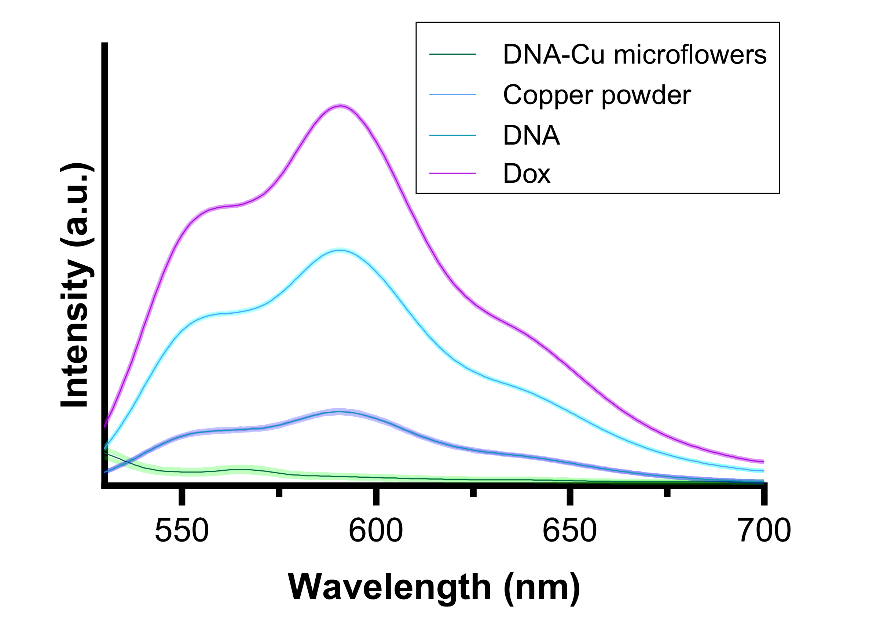


**Figure S13.** Fluorescence quantification of doxorubicin loading capacity (purple) comparing microflowers (0.1 mg, green), copper powder (1 mg, blue), and free DNA (0.1 μM, cyan). λ_ex_=494 nm, n=3.

**
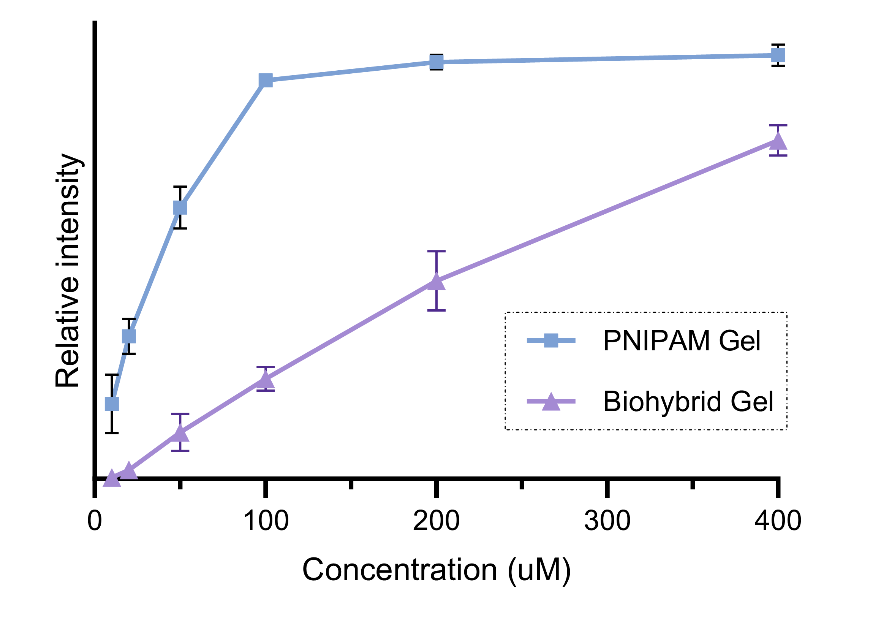
**

**Figure S14.** Fluorescence-based drug release comparing PNIPAM hydrogel (blue) and DNA-Cu MF biohybrid hydrogel (purple). Cumulative drug release data were collected under physiological conditions (pH 7.4, 37 ℃) over 2 hours.

**
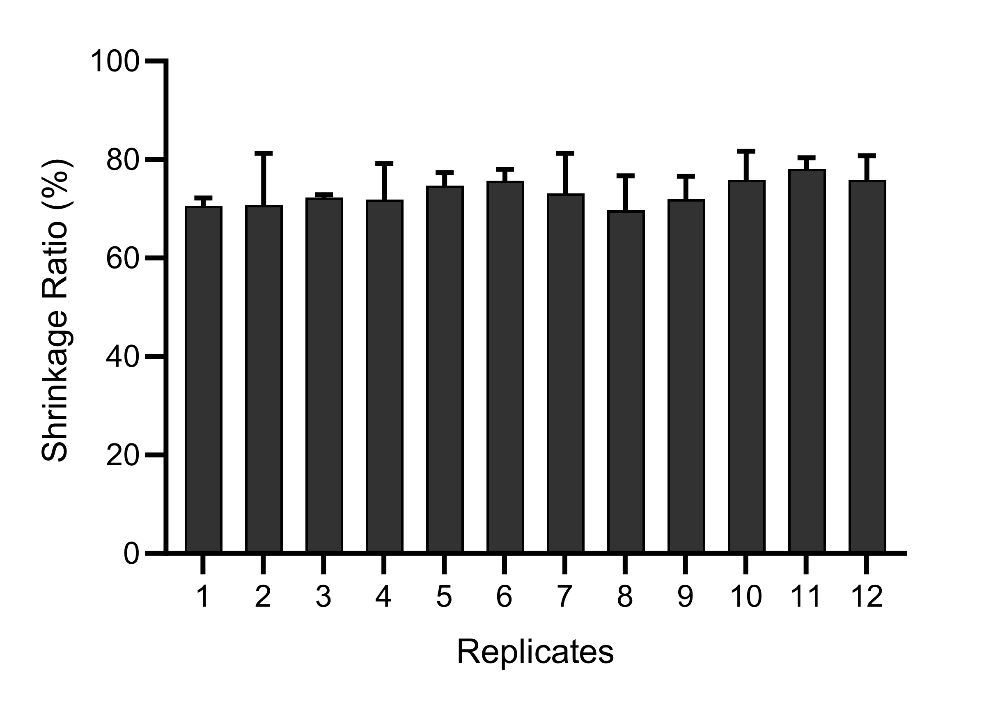
**

**Figure S15.** Volume shrinkage (%) of PNIPAM hydrogel under an AC voltage of 8 V over 12 repeated cycles (100 s per cycle).


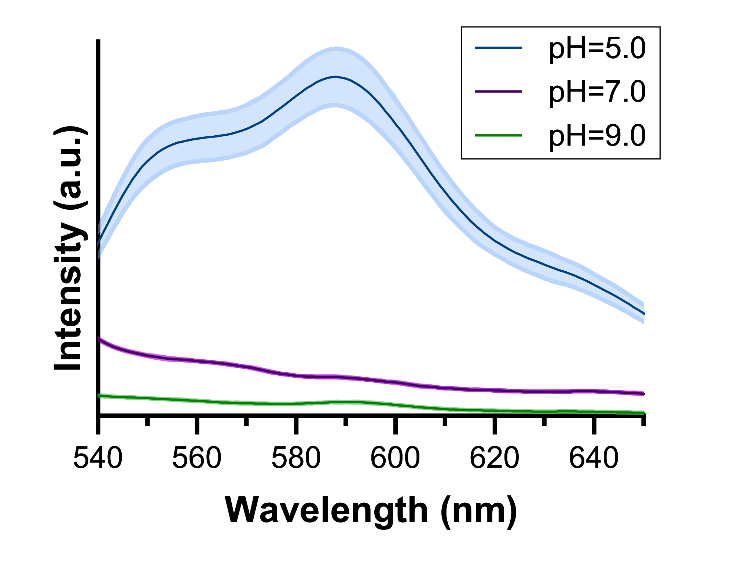


**Figure S16.** pH-dependent release of doxorubicin from microflower-loaded biohybrid hydrogels monitored by fluorescence intensity.

**
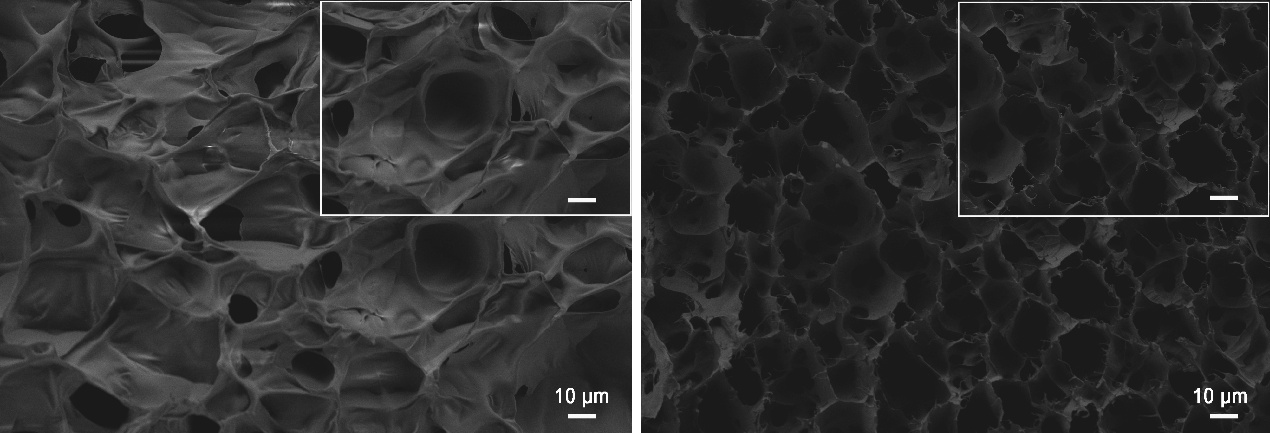
**

**Figure S17.** SEM images of the hydrogel before electrical stimulation and after multiple electrical cycles (scale bar: 10 μm).


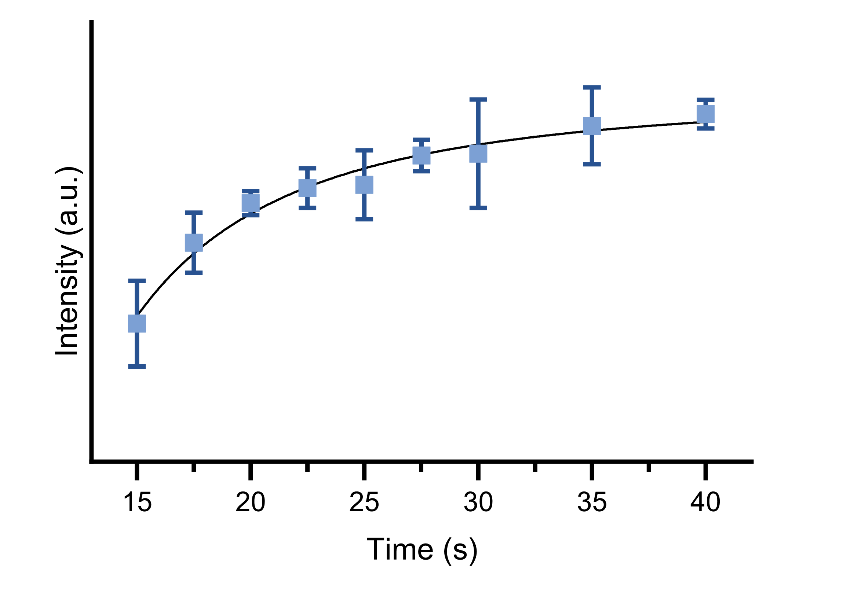


**Figure S18.** Real-time fluorescence intensity changes of drug released from the biohybrid hydrogel under 8 V AC stimulation.

**
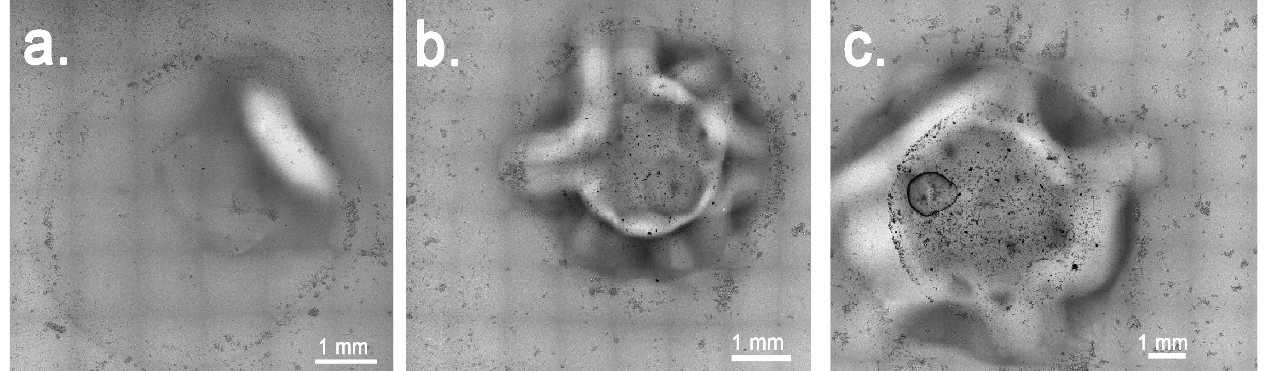
**

**Figure S19.** CLSM viability assessment of HeLa cells cultured with: (a) PNIPAM hydrogel, (b) Biohybrid hydrogel, and (c) Doxorubicin-loaded biohybrid hydrogel. Scale bars: 1 mm.

**
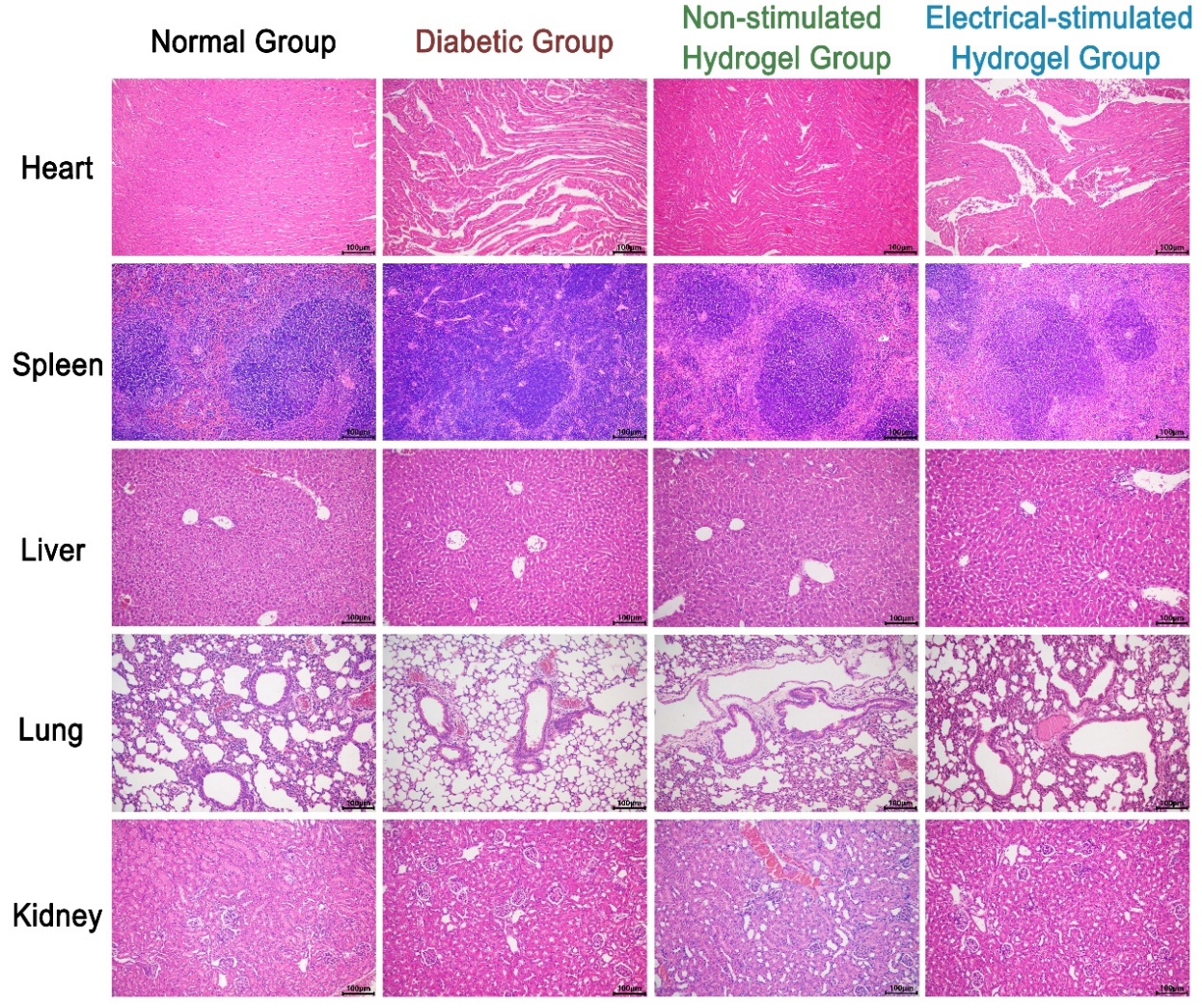
**

**Figure S20.** Histopathological evaluation of major organs (heart, liver, spleen, lung, kidney) by H&E staining. Scale bars: 100 μm.

**

**

**Figure S21.** SEM images of copper nanoflowers synthesized using copper powder (325 mesh, purity ≥99.5%) as the copper precursor.

**Table S2.** Hydrophobicity, loading and release performance of different model molecules.

| Drug | Loading Efficiency (%) | Release Efficiency (%) | Hydrophobicity (logP, approx.) |
| --- | --- | --- | --- |
| Nile Red | 77.81±5.38 | 43.32±14.94 | ~3-5 |
| Camptothecin | 83.92±2.69 | 77.41±8.24 | ~2.5-3.0 |
| Curcumin | 58.09±10.68 | 22.18±1.05 | ~3.0 |
| Daunorubicin | 64.67±2.02 | 64.55±0.72 | ~1.5-2.0 |
| Doxorubicin | 74.67±1.85 | 32.72±3.97 | ~1.3 |
